# Lipid-nanoparticle-mediated base editing of the trabecular meshwork rescues glaucoma in vivo

**DOI:** 10.1172/jci.insight.195593

**Published:** 2026-02-09

**Authors:** Balasankara Reddy Kaipa, Linya Li, Prakadeeswari Gopalakrishnan, Samuel Du, Jiin Felgner, Krzysztof Palczewski, Philip Felgner, Gulab S. Zode

**Affiliations:** 1 Gavin Herbert Eye Institute-Center for Translational Vision Research, Department of Ophthalmology,; 2 Department of Physiology and Biophysics, University of California, Irvine School of Medicine, Irvine, California, USA.

**Keywords:** Genetics, Ophthalmology, Gene therapy, Genetic diseases, Protein misfolding

## Abstract

Mutations in *MYOC*, the most common genetic cause of glaucoma, cause misfolded myocilin to accumulate in the endoplasmic reticulum (ER), leading to trabecular meshwork (TM) dysfunction, elevated intraocular pressure, and progressive vision loss. While gene editing offers curative potential, current delivery methods rely on viral vectors, which are limited by inflammation, off-target effects, and poor translatability. Here, we report a nonviral lipid nanoparticle (LNP) platform that enables selective in vivo delivery of mRNA encoding an adenine base editor and single guide RNA (LNP-ABE) to TM cells. A direct comparison of LNP-mCherry with lentiviral GFP revealed that LNPs outperform viral vectors, achieving markedly higher efficiency and greater selectivity for the TM without inducing ocular inflammation. In a Cre-inducible *Tg.CreMYOC^Y437H^* glaucoma mouse model, LNP-Cre mRNA selectively induced mutant *MYOC* expression in the TM, faithfully recapitulating key disease features. A single administration of LNP-ABE achieved efficient on-target editing of mutant *MYOC*, reducing mutant myocilin protein by approximately 46%, decreasing aggregates, alleviating ER stress, and fully rescuing the glaucomatous phenotype in *Tg.CreMYOC^Y437H^* mice. Importantly, no off-target editing or ocular toxicity was detected. These findings establish LNP-based mRNA delivery as a safe, efficient, and clinically translatable approach for TM-targeted genome editing with broad therapeutic potential in glaucoma.

## Abstract 2

A one-time gene-editing treatment using lipid nanoparticles safely targets eye tissue to correct a genetic cause of glaucoma and prevent vision loss in mice.

## Graphical abstract

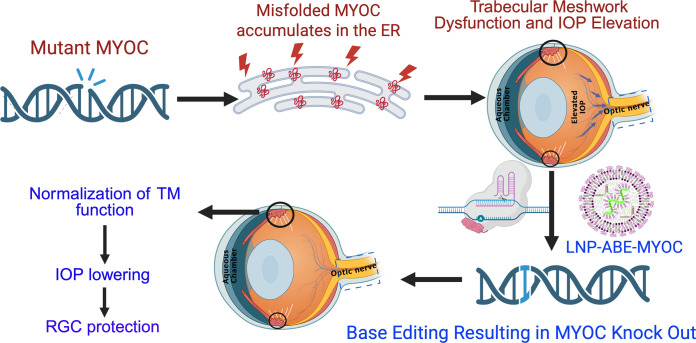

## Introduction

Glaucoma, the second leading cause of irreversible blindness globally, is characterized by the degeneration of retinal ganglion cells (RGCs), leading to irreversible vision loss (1, 2). Primary open-angle glaucoma (POAG) is the most common form of the disease, accounting for approximately 70% of all cases (2). POAG is associated with elevated intraocular pressure (IOP) due to damage to the trabecular meshwork (TM), a connective tissue critical for regulating aqueous humor outflow (3). Elevated IOP can result in the loss of RGCs and irreversible blindness (4). Current therapeutic approaches primarily focus on lowering IOP but fail to directly address TM pathology, leading to diminished efficacy over time. Over 10% of individuals with glaucoma ultimately become bilaterally blind due to a lack of targeted therapy (5). Targeting the molecular mechanisms underlying TM pathology presents a promising avenue for developing precision therapeutics for glaucoma.

Mutations in the myocilin gene (*MYOC*) represent a major genetic contributor to POAG, particularly in cases of juvenile-onset open-angle glaucoma, which typically manifests as high IOP in young children and progresses rapidly to vision loss (6, 7). Such cases often prove to be less responsive to existing treatments that fail to address the underlying pathology. While wild-type (WT) MYOC is not required for IOP regulation, mutations induce a deleterious gain of function. This leads to MYOC accumulation in the endoplasmic reticulum (ER), which triggers ER stress and TM cell death, elevating IOP and causing irreversible vision loss (7–15). Given that WT MYOC is not essential for IOP regulation (16) and that mutations result in harmful gain-of-function effects (17), knockout of *MYOC* via genome editing presents an ideal strategy for targeting glaucomatous pathways in the TM, potentially offering a one-time “cure” by restoring normal TM function. Using viral vectors, we have previously shown that targeting *MYOC* with the CRISPR/Cas9 system decreases mutant MYOC in the TM and prevents glaucoma in transgenic mice (18, 19). However, the traditional CRISPR/Cas9 delivery system posed 2 major challenges: ineffective tissue-specific delivery methods and significant off-target effects (20, 21). Viral vectors can lead to prolonged Cas9 expression, increasing the risk of nonspecific off-target effects and potential oncogenesis due to viral DNA integration (22–25).

To address these challenges, we set out to leverage recent advances in lipid nanoparticle (LNP) delivery systems to target Cas9 mRNA and single guide RNA (sgRNA) selectively to the TM while minimizing off-target effects. LNP-mRNA platforms offer several advantages, including low immunogenicity, high tropism based on the phagocytic nature of the TM, and fewer off-target effects than traditional viral vectors. To reduce off-target effects, we utilized base editors, which consist of a catalytically inactive Cas9 nuclease fused to a deaminase enzyme. Adenine base editors (ABEs) enable the conversion of A•T to G•C with high precision and efficiency without causing DNA double-strand breaks (21, 26, 27). This method can effectively knock out genes by altering key sequences, such as the initiation codon. We first conducted a direct comparison of LNPs and commonly used viral vectors (adeno-associated virus 2 [AAV2] and lentivirus [LV]) for in vivo delivery of reporter genes (mCherry, GFP, and Cre) to the TM. LNPs consistently outperformed viral vectors, achieving broad and selective TM transduction with markedly higher efficiency, while avoiding off-target ocular transduction and inflammation observed with viral delivery. Building on this delivery advantage, we engineered LNPs to encapsulate mRNA encoding an ABE and an sgRNA targeting the *MYOC* initiation codon (ATG→GTG) to disrupt translation of the mutant protein, and investigated whether this approach could efficiently deliver therapeutic mRNA to the TM in vivo, achieve precise *MYOC* knockout via base editing, and rescue glaucomatous phenotypes without inducing off-target editing or ocular toxicity.

## Results

### LNPs outperform viral vectors in TM tropism and safety.

**Figure 1:**
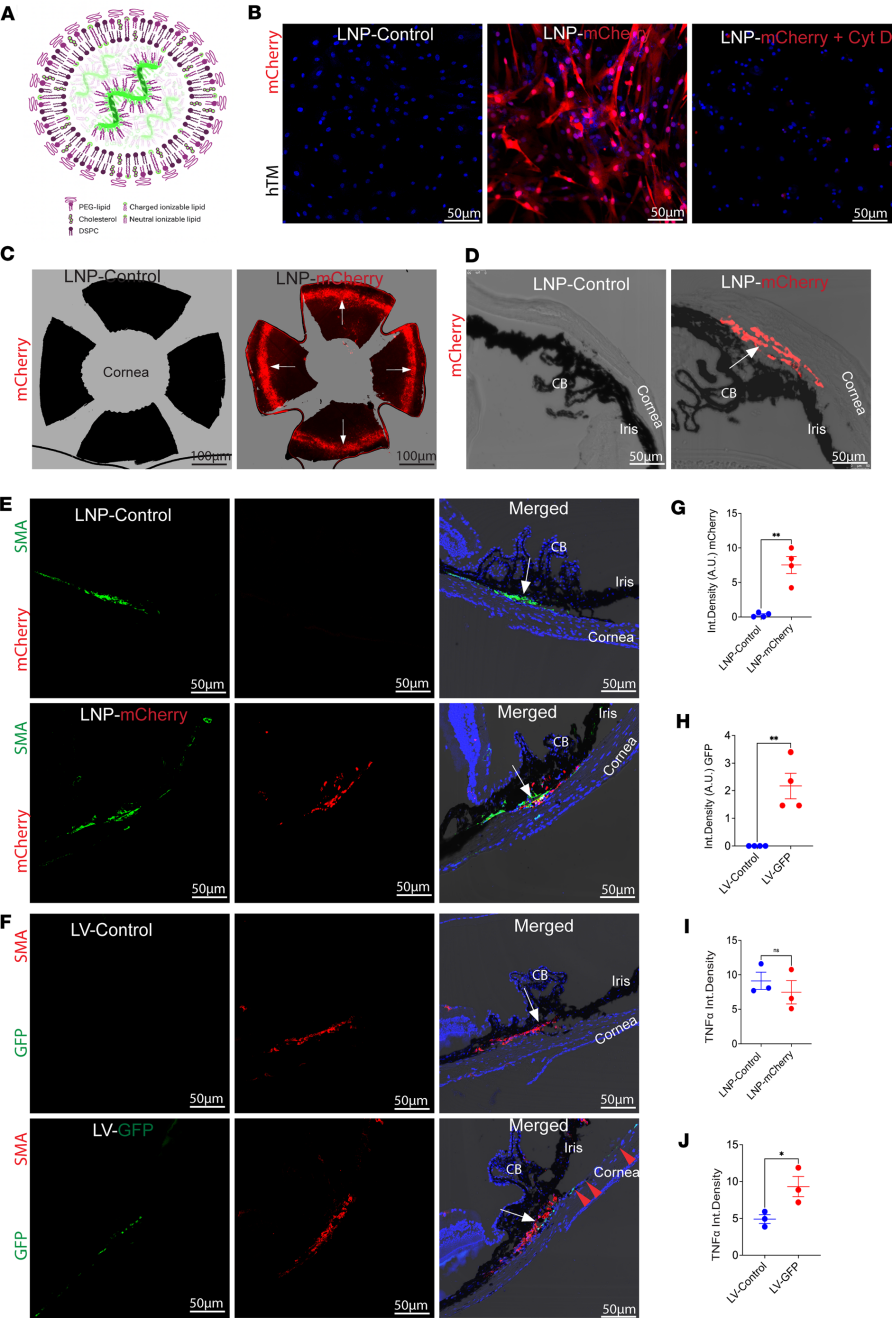
LNPs selectively and efficiently transduce the TM in vitro and in vivo, outperforming LV vectors. (**A**) Schematic of the LNP formulation containing ionizable lipids, cholesterol, DSPC, and PEG-lipids encapsulating mRNA. (**B**) Primary human TM (hTM) cells treated with LNP-mCherry exhibited robust mCherry expression, which was abolished by cytochalasin D (Cyt D), indicating uptake via phagocytosis. *n* = 3 donors. (**C** and **D**) Intracameral (IC) injection of LNP-mCherry (1.4 μg/eye) in C57BL/6J mice produced strong mCherry expression selectively in the TM, as shown in flat-mount anterior segments (**C**) and cross sections (**D**). (**E**) Immunostaining with α-SMA (a marker for TM and ciliary bodies) confirmed that mCherry expression was localized to the TM in LNP-mCherry–treated eyes but absent in controls. (**F**) Comparative analysis with LV showed limited TM transduction and off-target expression in corneal endothelium, whereas LNPs produced robust and selective TM expression. (**G** and **H**) Quantification demonstrated that LNPs achieved significantly higher TM expression of the reporter gene (mCherry, **G**) compared with LV (GFP, **H**), **P = 0.0032 (unpaired 2-tailed *t* test). (**I** and **J**) Immunostaining analysis revealed no increase in TNF-α expression following LNP-mCherry delivery, whereas LV-GFP induced a significant inflammatory response. Data are presented as mean ± SEM; *n* = 3–4 mice per group. *P = 0.0430 (unpaired 2-tailed *t* test). NS, not significant; CB, ciliary body. Arrows indicate the location of the TM. Scale bars: 50 μm.

To determine whether LNPs can deliver mRNA to the TM cells in vitro and in vivo, we first encapsulated mCherry mRNA in LNPs formulated with commercially available lipids, including 1,2-distearoyl-*sn*-glycero-3-phosphocholine 280 (DSPC), cholesterol, and 1,2-dimyristoyl-rac-glycero-3-methoxypolyethylene glycol-2000 (DMG-281 PEG 2000-DMG) (Figure 1A and Supplemental Figure 1A; supplemental material available online with this article; https://doi.org/10.1172/jci.insight.195593DS1). mRNA modified with 5-methoxyuridine (MOU) was used to enhance stability and lower immunogenicity, and particle size and encapsulation efficiency were assessed to ensure the uniformity and quality of each LNP formulation (Supplemental Figure 1, B and C).

In primary human TM cells (*n* = 3 strains), LNP–mCherry mRNA (1.8 μg/mL) induced robust mCherry expression within 24 hours, with nearly 100% of cells transfected. LNP uptake was significantly inhibited by cytochalasin D, an inhibitor of phagocytosis (28), indicating that TM cells most likely internalize LNPs via phagocytosis (Figure 1B and Supplemental Figure 1D). We next evaluated in vivo delivery. Intracameral (IC) injection of LNP–mCherry mRNA (1.4 μg/eye) into C57BL/6J mice produced strong mCherry expression throughout the TM within 48 hours, as observed in flat mounts and cross sections (Figure 1, C and D). Importantly, no mCherry signal was detected in non-TM ocular cells, confirming selective targeting (Supplemental Figure 1, E and F). Note that LNP–mCherry mRNA experiments were assessed at 48 hours, as mCherry protein expression declines within 2–4 days.

We next compared LNPs with 2 commonly used viral vectors, AAV2 and LV. Previously, we have shown that AAV2-GFP exhibits poor transduction of TM and primarily targets RGCs, whereas LV-GFP preferentially transduces TM and the corneal endothelium (19). We first compared reporter gene expression in the TM of LNP-mCherry (1.4 μg mRNA/eye) or LV-GFP (2.5 × 10^^6^^ transducing units [TU]/eye) injected via the IC route. Immunostaining for α-smooth muscle actin (α-SMA) was used to identify the TM region, although α-SMA is also expressed in ciliary bodies, as no selective TM marker exists. LNP-mCherry produced strong, uniform TM expression within 48 hours (Figure 1E), whereas LV-GFP produced only limited TM signal after 2 weeks (Figure 1F) and showed prominent expression in corneal endothelium and retinal pigmented epithelium (Supplemental Figure 1, G and H). Quantification of reporter gene integrated density revealed that LNP-mCherry achieved more efficient delivery to the TM compared with LV (Figure 1, G and H). Low-magnification images further demonstrated that LNPs did not transduce non-TM ocular cells, including RGCs (Supplemental Figure 1, E and F), while LV-GFP transduced corneal endothelium and retinal pigmented epithelium (Supplemental Figure 1, G and H). To assess inflammatory responses, we examined TNF-α and GFAP expression following delivery. LV-GFP induced significant TNF-α upregulation in the outflow pathway and increased GFAP in the RGC layer, whereas LNP-mCherry had no effect on either marker (Figure 1, I and J, and Supplemental Figure 2, A–F).

**Figure 2:**
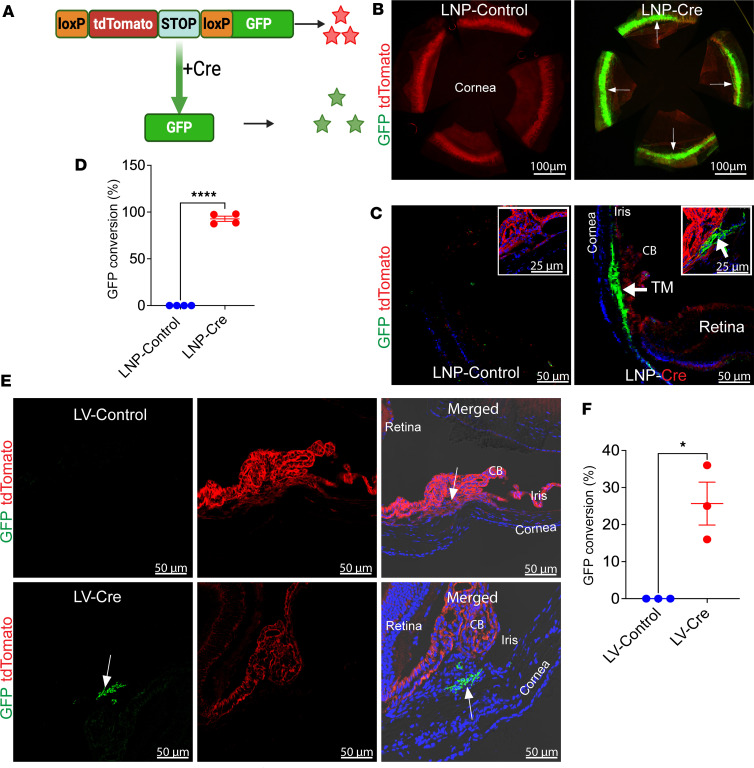
LNP-Cre induces efficient and selective Cre recombination in the TM of mTmG reporter mice, outperforming LV vectors. (**A**) Schematic of the mTmG reporter mice: All cells express tdTomato (red) until Cre-mediated recombination removes the STOP cassette, switching expression to GFP (green). (**B**) Flat-mount anterior segments showing strong GFP expression throughout the TM of LNP-Cre–injected eyes, compared with tdTomato-only expression in controls. (**C**) Cross sections confirmed robust GFP induction specifically in the TM region of LNP-Cre–treated mice (arrow), whereas controls retained tdTomato. Insets highlight TM conversion. (**D**) Quantification of GFP conversion demonstrated nearly complete (~100%) recombination in the TM following LNP-Cre, compared with no conversion in controls (*n* = 4 per group; ****P < 0.0001, unpaired 2-tailed *t* test). (**E**) In contrast, LV-Cre injection produced patchy and inefficient GFP expression in the TM (arrow), with predominant tdTomato expression persisting in surrounding tissues. (**F**) Quantification of GFP intensity confirmed significantly lower recombination efficiency in LV-Cre–treated mice compared with LNP-Cre (*n* = 4; *P = 0.0193, unpaired 2-tailed *t* test). CB, ciliary body; Arrows indicate the TM location. Scale bars: 100 μm (**B**) and 50 μm (**C** and **E**).

We evaluated the functional activity of different delivery approaches by comparing LNP-Cre, AAV2-Cre, and LV-Cre in mTmG reporter mice (Figure 2). In this system, all cells initially express tdTomato (red), which switches to GFP (green) following Cre-mediated recombination (Figure 2A). A single IC injection of LNP-Cre (1.4 μg/eye) produced near-complete recombination throughout the TM, with robust GFP expression detectable in both flat mounts (Figure 2B) and cross sections (Figure 2C). Quantification confirmed approximately 100% GFP-positive TM cells by 2 weeks after injection (Figure 2D). No GFP signal was detected in the retina or other ocular tissues for LNP-Cre (Supplemental Figure 3A). In contrast, AAV2-Cre and LV-Cre induced lower and more patchy TM recombination, with LV-Cre achieving only approximately 25% GFP conversion compared with nearly 100% in LNP-Cre–injected eyes (Figure 2, E and F, and Supplemental Figure 3, B–D). Together, these findings demonstrate that LNPs deliver functional mRNA to the TM with markedly higher efficiency and uniformity than viral vectors, while maintaining excellent tissue selectivity. This superior tropism positions LNPs as a promising platform for TM-targeted genome editing therapies.

### A single IC injection of LNP-Cre induces mutant MYOC in the TM, leading to glaucoma in a Cre-inducible mouse model of MYOC-associated POAG.

**Figure 3:**
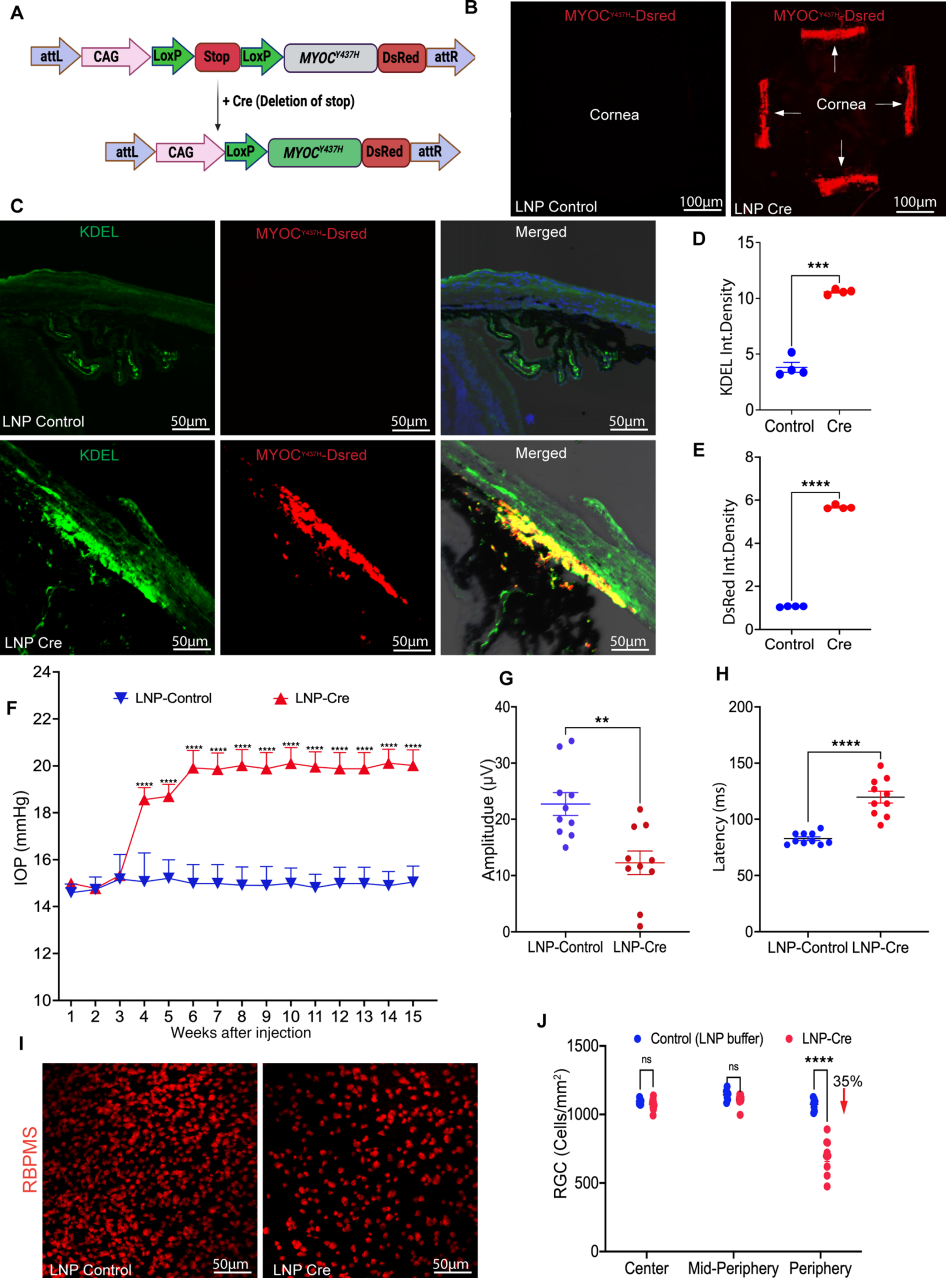
LNP-Cre mRNA induces glaucoma in a Cre-inducible *MYOC* glaucoma mouse model. (**A**) Schematic design of *Tg.CreMYOC^Y437H^* mice. A STOP cassette prevents transgene expression until Cre excision induces expression of mutant MYOC. *Tg.CreMYOC^Y437H^* mice were injected IC with LNP-Cre mRNA, and glaucoma phenotypes were assessed. (**B**) Flat-mount anterior segments showing robust DsRed-MYOC expression throughout the TM in LNP-Cre–treated eyes compared with the controls. (**C**) Immunostaining for ER marker KDEL revealed colocalization with mutant MYOC, indicating ER stress in LNP-Cre–treated eyes. (**D** and **E**) Quantification of KDEL and DsRed fluorescence intensities confirmed significant increases in LNP-Cre–treated mice compared with controls (*n* = 4 per group; ***P = 0.0004; ****P < 0.0001; unpaired 2-tailed *t* test). (**F**) IOP measurements showed sustained ocular hypertension beginning at week 4 after injection in LNP-Cre–treated eyes compared with controls (*n* = 24 LNP-Cre, *n* = 20 LNP-buffer; ****P < 0.0001; 2-way ANOVA with Šídák’s multiple comparisons test). (**G** and **H**) PERG revealed reduced amplitudes and increased latencies in LNP-Cre–treated mice, indicating significant RGC functional loss (*n* = 10 per group; **P = 0.0021; ****P < 0.0001; unpaired 2-tailed *t* test). (**I** and **J**) Representative RBPMS staining of retinal flat mounts and quantification showed significant RGC loss in the peripheral retina of LNP-Cre–treated eyes (35% reduction), while central and mid-peripheral regions were unaffected (*n* = 10 LNP-Cre, *n* = 8 controls; ****P < 0.0001; 2-way ANOVA with Šídák’s multiple comparisons). NS, not significant; TM, trabecular meshwork; CB, ciliary body. Scale bars: 100 μm (**B**) and 50 μm (**C** and **I**).

We have recently developed a Cre-inducible transgenic mouse that expresses the DsRed-tagged Y437H mutant of human *MYOC* (referred to as *Tg.CreMYOC^Y437H^*) (29). Under normal conditions, mutant human *MYOC* is not expressed. The expression of Cre recombinase leads to the removal of the STOP cassette, resulting in the expression of DsRed-fused mutant *MYOC* (Figure 3A). We have recently demonstrated that intravitreal injection of helper adenovirus 5 (HAd5) induced mutant MYOC in the TM, leading to significant IOP elevation and RGC loss in *Tg.CreMYOC^Y437H^* mice (29). However, HAd5-Cre was not specific to TM cells, and it induced mutant MYOC in the corneal endothelium as well. Since LNPs exhibited specific tropism to TM cells, we sought to examine whether LNP-Cre mRNA would induce mutant MYOC selectively in the TM, leading to IOP elevation and RGC loss in *Tg.CreMYOC^Y437H^* mice (Figure 3). Six-month-old *Tg.CreMYOC^Y437H^* mice were injected IC with LNP–Cre mRNA (1.4 μg/mL). Confocal microscopy of DsRed protein in flat-mount anterior segments (Figure 3B) revealed that mutant MYOC is induced selectively in the entire TM region after LNP–Cre mRNA injection in *Tg.CreMYOC^Y437H^* mice. Mutations in *MYOC* lead to protein misfolding, causing its accumulation in the ER. Immunostaining for the ER marker KDEL demonstrated a robust increase in KDEL, which strongly colocalized with DsRed-fused MYOC selectively in the TM region (Figure 3C). Quantification of fluorescence demonstrated a significant increase in MYOC and KDEL in the TM of Cre-injected *Tg.CreMYOC^Y437H^* mice (Figure 3, D and E). IOP measurements further revealed that expression of the mutant MYOC gene by LNP–Cre mRNA elevated IOP significantly in *Tg.CreMYOC^Y437H^* mice starting from 4 weeks after injection, and IOP remained elevated throughout the course of the study (Figure 3F). Notably, sustained IOP elevation led to functional loss of RGCs, as evidenced by significantly decreased amplitudes in the pattern electroretinogram (PERG) and increased latencies in *Tg.CreMYOC^Y437H^* mice 15 weeks after LNP–Cre mRNA injection (Figure 3, G and H, and Supplemental Figure 4, A and B). Immunostaining of retinal flat mounts for the RGC marker, RNA-binding protein with multiple splicing (RBPMS), showed a significant loss of RGCs in the periphery of the retina in *Tg.CreMYOC^Y437H^* mice 15 weeks after LNP–Cre mRNA injection (Figure 3, I and J, and Supplemental Figure 4C). These data indicate that a single injection of LNP–Cre mRNA induces mutant MYOC expression selectively in the TM, leading to glaucoma in *Tg.CreMYOC^Y437H^* mice.

### LNP–ABE mRNA edits MYOC and prevents intracellular accumulation of MYOC in cultured TM cells.

**Figure 4:**
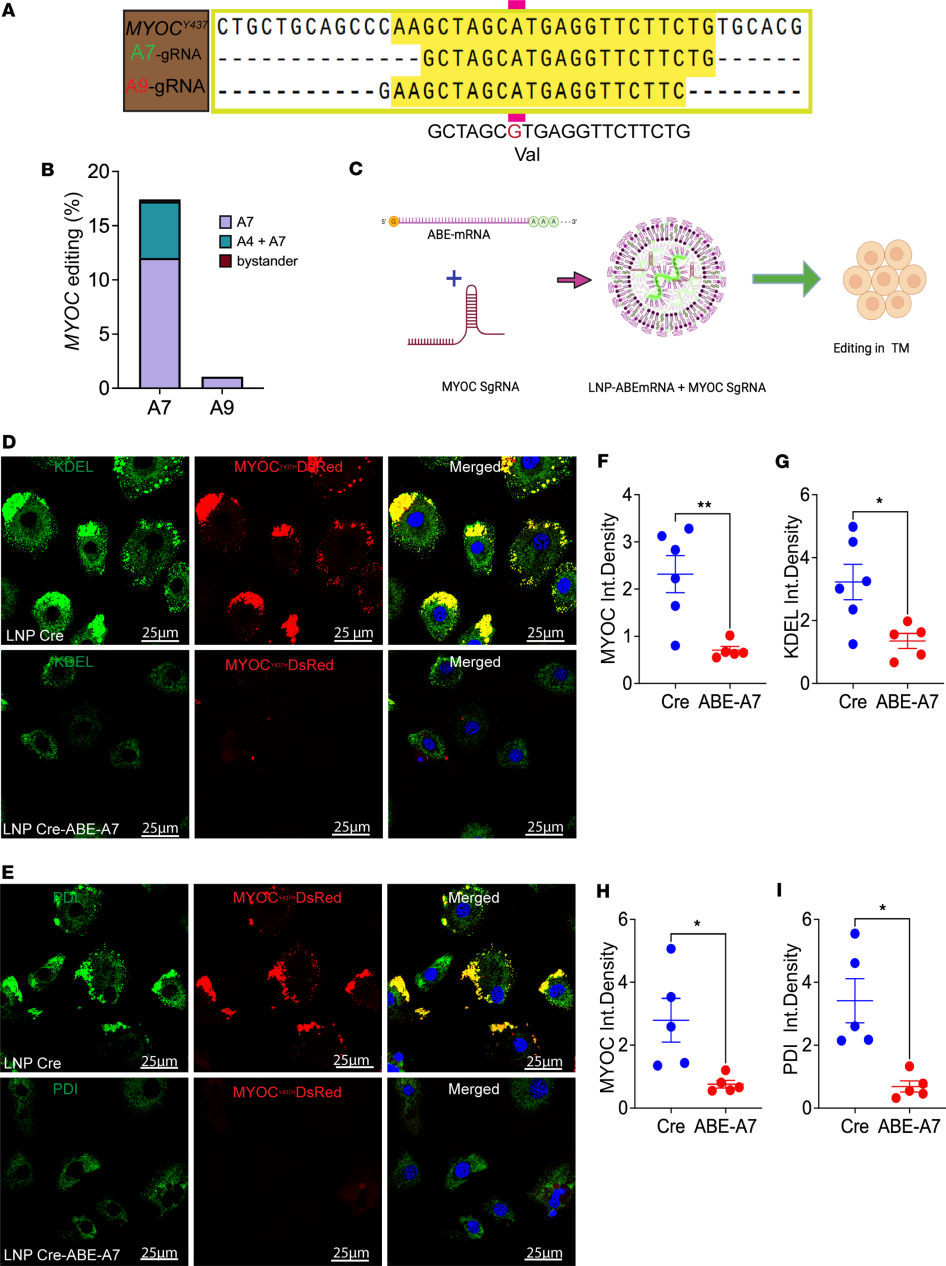
LNP-ABE edits *MYOC* and reduces intracellular accumulation of mutant MYOC in TM cells. (**A**) Schematic of sgRNA design targeting the *MYOC* initiation codon (ATG→GTG) to disrupt translation. Two sgRNAs (A7 and A9) were tested. (**B**) Editing efficiencies in mutant *MYOC*–expressing mouse fibroblasts transfected with ABE plasmids showed robust activity of A7 sgRNA compared with A9. (**C**) Experimental design: LNPs were formulated with ABE mRNA and MYOC-targeting sgRNA for delivery to TM cells. (**D** and **E**) Outflow cells from the iridocorneal angle of *Tg.CreMYOC^Y437H^* mice were treated with LNP-Cre or LNP-Cre+LNP-ABE-A7. Immunostaining demonstrated reduced intracellular MYOC-DsRed accumulation and decreased ER stress markers (KDEL and PDI) following ABE A7 treatment. (**F**–**I**) Quantification of fluorescence intensity confirmed significant reductions in MYOC-DsRed, KDEL, and PDI levels in LNP-ABE-A7–treated TM cells compared with controls (*n* = 5–6 per group; *P < 0.05, **P < 0.01; unpaired 2-tailed *t* test). Scale bars: 25 μm.

Next, we sought to examine whether LNPs encapsulating ABE mRNA and sgRNA targeting *MYOC* mediate effective editing of the gene and decrease the intracellular accumulation of MYOC in vitro. We utilized ABE to knock out *MYOC* by altering the initiation codon (ATG to GTG). We designed 2 sgRNAs (A7 and A9) to target *MYOC* (Figure 4A and Supplemental Table 1). To determine which sgRNA targets *MYOC* more efficiently, mouse primary fibroblast cells expressing human mutant MYOC were transduced for 72 hours with plasmids expressing ABE sgRNA A7 or A9. Next-generation sequencing (NGS) analysis demonstrated that A7 sgRNA targets MYOC with 17% efficiency, whereas A9 sgRNA was ineffective in editing *MYOC* (Figure 4B). Based on these results, sgRNA A7 was selected for LNP formulation. It should be noted that low editing efficiency is likely due to the low transfection efficiency of plasmids in primary mouse fibroblasts.

Next, ABE mRNA (ABE8e-SpCas9-NG) was synthesized using in vitro transcription, and the plasmid map showed correct gene amplification (Supplemental Figure 5A). To further ensure the quality of the ABE mRNA, it was analyzed via agarose gel electrophoresis, which showed a single band, documenting pure ABE mRNA (Supplemental Figure 5B). Indeed, bioanalyzer data indicated high purity of the ABE mRNA (Supplemental Figure 5C). We utilized commercially available ionizable lipids, including the ionizable cationic lipid ALC0315, combined with helper lipids DSPC, cholesterol, and PEG2000-DMG at a molar ratio of 50:10:38.5:1.5, respectively, to formulate LNP–ABE mRNA and sgRNA targeting *MYOC*, as shown in the schematic design in Figure 4C. Dynamic light scattering demonstrated that the LNPs were monodisperse, with a hydrodynamic diameter of approximately 70 nm (Supplemental Figure 5D). Encapsulation efficiency was observed to be almost 95% (Supplemental Figure 5E). We next documented that LNP–ABE mRNA produces Cas9 protein in GTM3 cells by Western blot analysis. The presence of Cas9 protein was evident 24 hours after adding the LNPs but was noticeably decreased after 48 hours (Supplemental Figure 5F). Next, we examined whether LNPs carrying ABE mRNA and sgRNA A7 (ABE-A7) could knock out mutant *MYOC* and prevent ER stress in cultured mouse outflow cells (cultured from the iridocorneal angle tissue of *Tg.CreMYOC^Y437H^* mice). Cultured outflow cells were incubated with LNPs carrying Cre mRNA (to induce mutant MYOC) or with Cre mRNA plus ABE-A7 for 72 hours. Immunostaining for ER stress marker, KDEL, and PDI, and its analyses demonstrated that LNP–Cre mRNA induced mutant MYOC in TM cells, and treatment with LNP-ABE-A7 significantly reduced intracellular accumulation of mutant MYOC and its associated ER stress markers (Figure 4, D–I).

### LNP-ABE edits MYOC and reduces its intracellular accumulation in mouse TM in vivo.

**Figure 5:**
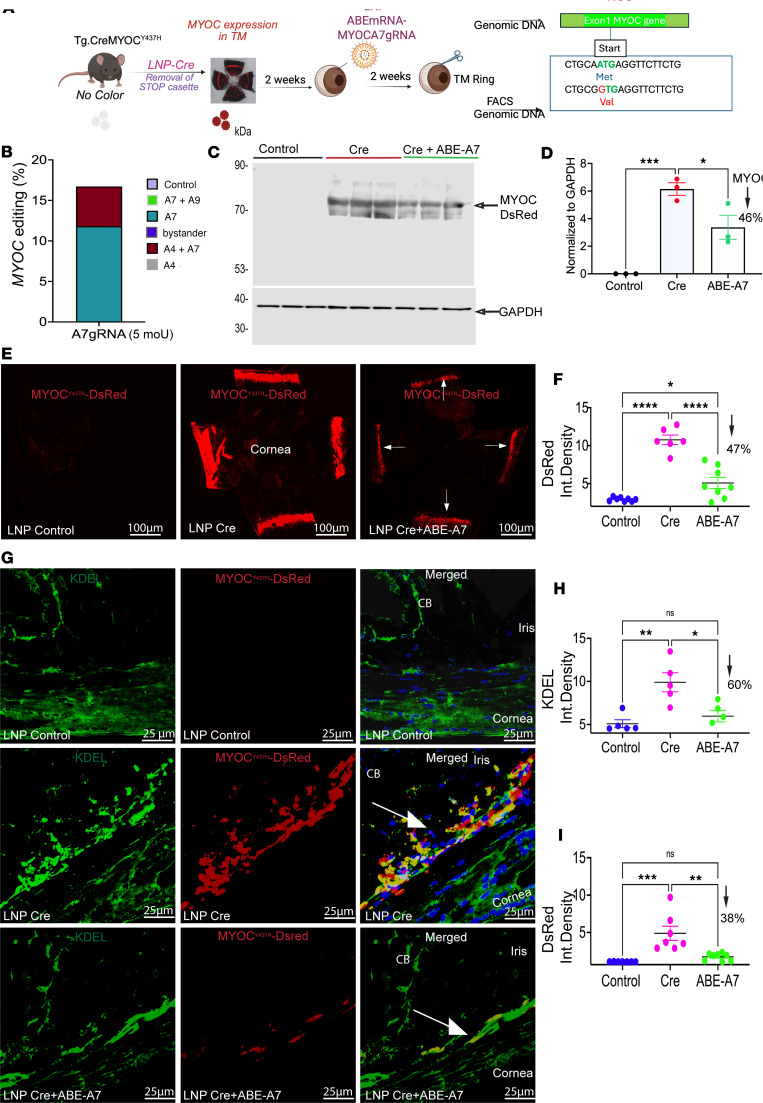
LNP-ABE-A7 edits *MYOC* and reduces intracellular mutant protein accumulation in the TM of *Tg.CreMYOC^Y437H^* mice. (**A**) Experimental design. Adult *Tg.CreMYOC^Y437H^* mice were injected IC with LNP-Cre to induce mutant *MYOC* expression in the TM. Two weeks later, mice received a second IC injection of LNPs carrying either buffer or ABE-A7. After 2 weeks, TM rings were dissected for NGS and immunostaining. (**B**) NGS of FACS-isolated TM cells from the iridocorneal angle revealed approximately 17% *MYOC* editing in ABE-A7–treated mice compared with controls (*n* = 6 eyes pooled together for LNP-ABE-A7 and the controls, respectively). (**C** and **D**) Western blot analysis of DsRed-MYOC protein in TM tissues confirmed an approximately 46% reduction in mutant MYOC following ABE-A7 treatment. Quantification of protein levels is shown in **D**. *n* = 3 eyes per group, Ordinary 1-way ANOVA followed by Dunnet’s test, *P = 0.0240, ***P = 0.0005. (**E** and **F**) Flat-mount imaging and quantification showed that ABE-A7 treatment reduced DsRed-MYOC accumulation in the TM by approximately 47% compared with Cre-injected controls (*n* = 7–8 per group; ordinary 1-way ANOVA followed by Tukey’s test), ****P < 0.0001). (**G**–**I**) Representative immunostaining of anterior segment cross sections for ER stress marker (KDEL) revealed colocalization with mutant MYOC in Cre-injected eyes. ABE-A7 treatment significantly reduced KDEL (~60%) and DsRed-MYOC (~38%) intensity compared with Cre controls (*n* = 5–7 per group; ordinary 1-way ANOVA followed by Tukey’s test), *P < 0.05; **P < 0.01; ***P < 0.001). Arrows indicate the TM location. NS, not significant; CB, ciliary body. Scale bars: 100 μm (**E**) and 25 μm (**G**).

We next explored whether LNP-ABE-A7 edits *MYOC* and prevents its accumulation in the TM of *Tg.CreMYOC^Y437H^* mice. Expression of mutant MYOC in the TM was induced in adult *Tg.CreMYOC^Y437H^* mice via IC injection of LNP-Cre. Two weeks after injection, LNPs carrying ABE-A7 (1.4 μg/mL) or LNPs with buffer (LNP-buffer) were injected via the IC route. The iridocorneal angle was dissected, and genomic DNA was analyzed by NGS to determine editing efficiency (Figure 5A). Because this tissue includes TM, ciliary bodies, sclera, and corneal endothelium, we further digested the tissue with papain and isolated DsRed-positive TM cells by fluorescence-activated cell sorting (FACS), enabling selective analysis of TM editing.

We first assessed whether mRNA modifications affect *MYOC*-editing efficiency via NGS of outflow-pathway tissues. We compared the *MYOC*-editing efficiency of unmodified and MOU-modified ABE mRNA in the iridocorneal angle tissue (Supplemental Figure 5, G and H). LNPs encapsulating unmodified or MOU-modified ABE mRNA and sgRNA were injected IC into *Tg.CreMYOC^Y437H^* mice and iridocorneal angle tissues were analyzed by NGS 2 weeks after injection. We observed 3.5% editing efficiency with unmodified ABE mRNA (Supplemental Figure 5G), while incorporation of MOU modifications increased editing efficiency to 5.6% (Supplemental Figure 5H). Based on these findings, we utilized MOU-modified ABE mRNA for all subsequent studies.

It is important to note that these values likely underestimate true editing efficiency, as NGS was performed on whole iridocorneal angle tissue, which contains ciliary bodies, sclera, and other ocular cell types in addition to the TM cells. Since LNPs selectively target TM cells, the proportion of edited TM cells is expected to be substantially higher. To obtain editing from TM cells, we isolated the TM cells via FACS of DsRed-positive cells and performed NGS. The isolated TM cells exhibited 17% editing efficiency in *Tg.CreMYOC^Y437H^* mice treated with MOU-modified ABE mRNA (Figure 5B). This value likely underestimates true editing due to incomplete recovery of TM cells, and some edited TM cells may exhibit low DsRed expression, making them less likely to be captured.

To evaluate the functional consequences of editing, we measured DsRed-MYOC levels in the iridocorneal angle tissue. Because LNP-Cre–induced DsRed-MYOC expression was restricted to the TM, this allowed for the selective analysis of MYOC protein from the iridocorneal angle tissue (Figure 5, C–I). Western blot analysis confirmed an approximately 46% reduction in mutant MYOC protein in ABE-A7-*Tg.CreMYOC^Y437H^* mice (Figure 5, C and D). In addition, *MYOC*-editing-mediated reduction of mutant MYOC was associated with diminution of ER stress markers, including GRP78, GRP94, and CHOP (Supplemental Figure 5I). Flat-mount imaging of the anterior segment demonstrated robust induction of mutant DsRed-MYOC throughout the TM following LNP-Cre, which was reduced by 47% with LNP-ABE-A7 treatment in the TM of *Tg.CreMYOC^Y437H^* mice (Figure 5, E and F). Immunostaining of cross sections further corroborated these results (Figure 5, G–I). Finally, DsRed-MYOC and its colocalization with the ER marker KDEL demonstrated that LNP-Cre induced DsRed–mutant MYOC accumulation in the ER of mouse TM, which was significantly reduced by 38% in ABE-A7-*Tg.CreMYOC^Y437H^* mice (Figure 5, H and I). Together, these results demonstrate that LNPs carrying ABE-A7 efficiently edit *MYOC* in vivo, leading to significant reductions in mutant MYOC transcripts and protein, and decreasing its pathological accumulation in the ER of TM cells in *Tg.CreMYOC^Y437H^* mice.

### LNP-ABE rescues glaucoma in a mouse model of MYOC-associated glaucoma.

**Figure 6:**
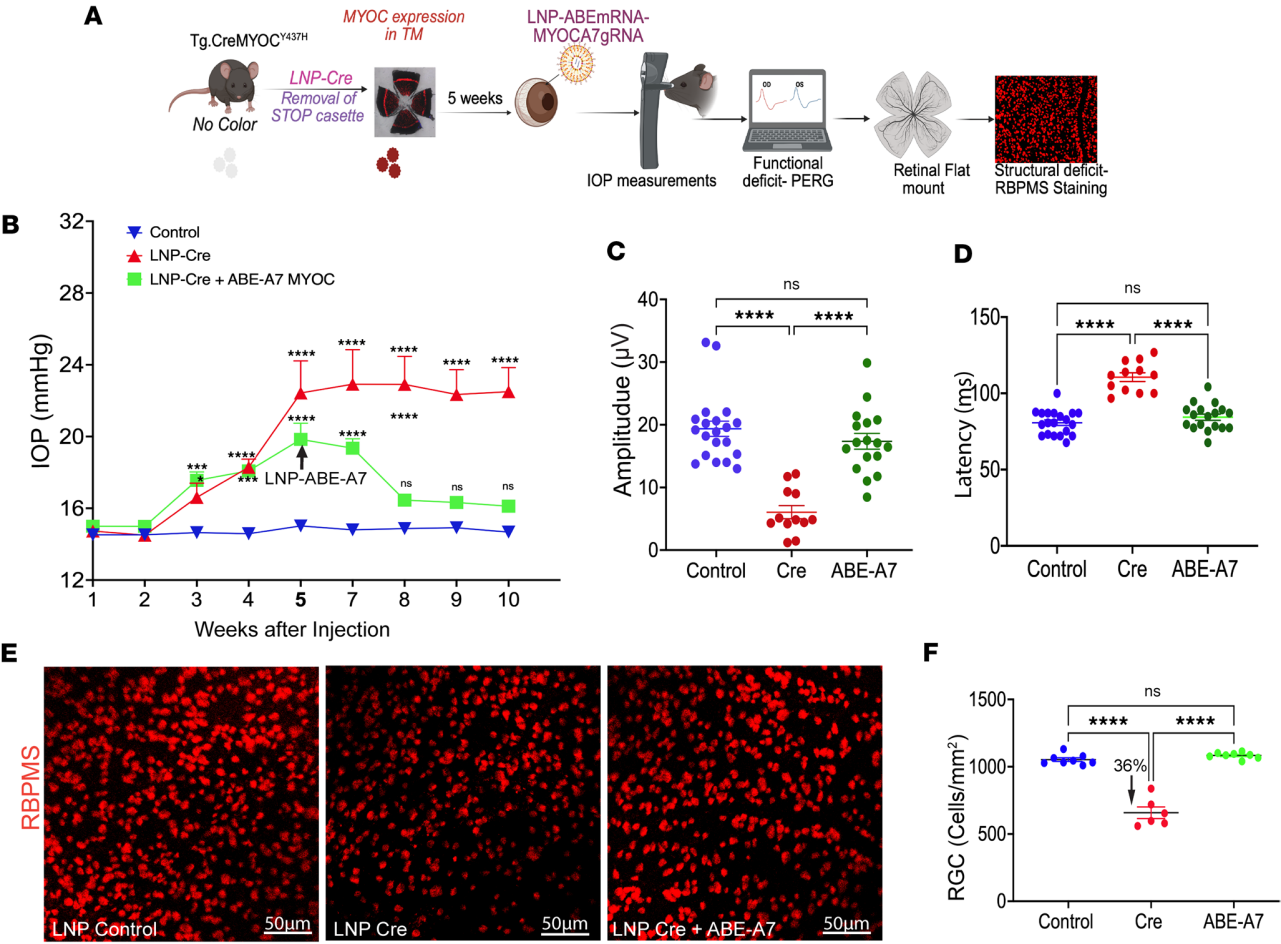
LNP-ABE-A7 rescues glaucoma in a mouse model of glaucoma. (**A**) Schematic of the study design. Six-month-old *Tg.CreMYOC^Y437H^* mice were injected with LNP-Cre to induce mutant MYOC expression in the TM, resulting in ocular hypertension. Five weeks later, Cre-induced mice were treated with IC injection of either LNP-ABE-A7 or LNP-buffer, and glaucoma phenotypes were evaluated. (**B**) Weekly IOP measurements demonstrated that *MYOC* editing via ABE-A7 decreased ocular hypertension in Cre-induced *Tg.Cre-MYOC^Y437H^* mice compared with controls (*n* = 16 for LNP-control, *n* = 12 for LNP-Cre, and *n* = 20 for ABE-A7; ****P < 0.0001; 2-way ANOVA followed by Fisher’s multiple comparison [LSD] test). (**C** and **D**) LNP-ABE-A7 treatment improved RGC function significantly in Cre-induced *Tg.CreMYOC^Y437H^* mice, as evident from improved PERG amplitude and decreased latency (*n* = 20 for LNP-control, *n* = 12 for LNP-Cre, and *n* = 18 for ABE-A7; ****P < 0.0001; ordinary 1-way ANOVA followed by Tukey’s test). (**E** and **F**) Representative flat-mount retina staining for RBPMS (**E**) and quantification of RGC density (**F**) revealed that ABE-A7 treatment prevented RGC loss, with approximately 36% higher survival compared with Cre-induced mice (*n* = 8 for LNP-control, *n* = 6 for LNP-Cre, and *n* = 8 for ABE-A7; ****P < 0.0001; ordinary 1-way ANOVA followed by Tukey’s test). Scale bars: 50 μm.

Six-month-old *Tg-CreMYOC^Y437H^* mice were first injected IC with LNP-Cre to induce mutant MYOC, and IOP was monitored for 5 weeks. Then, ocular-hypertensive *Tg-CreMYOC^Y437H^* mice were injected IC with LNP-ABE-A7 or control (LNP-buffer), and glaucoma phenotypes were examined (Figure 6A). IOP values in both LNP-Cre groups were significantly higher (****P < 0.0001) than in the LNP-buffer groups before ABE-A7 treatment, with no significant difference between the 2 LNP-Cre groups. (Figure 6B). Five weeks after injection of Cre, the ocular-hypertensive *Tg-CreMYOC^Y437H^* mice were divided randomly into 2 groups; 1 group received an IC injection of LNP-buffer, while the other group received LNPs carrying ABE-A7 (1.4 μg/eye). Following ABE-A7 administration, the ABE-A7+LNP-Cre group showed a significant reduction in IOP compared with the LNP-buffer+LNP-Cre group. There was no significant difference between the ABE-A7+LNP-Cre and the LNP-buffer groups after treatment, demonstrating effective normalization of IOP following base editing.

IOP measurements after 2 weeks revealed that treatment with LNP-ABE-A7 significantly decreased IOP compared with the control (Figure 6B). Notably, the diminution of IOP in the ABE-A7–treated mice was sustained throughout the course of the study. We next examined whether ABE-A7 prevented the functional and structural loss of RGCs in *Tg-CreMYOC^Y437H^* mice, using PERG (Figure 6, C and D) and RBPMS staining of whole-mount retinas (Figure 6, E and F). Fifteen weeks after LNP-Cre injection, ABE-A7–treated *Tg-CreMYOC^Y437H^* mice exhibited significantly improved PERG amplitudes and decreased latencies compared with control *Tg.CreMYOC^Y437H^* mice, indicating that ABE-A7 prevents functional loss of RGCs. Consistent with this observation, RBPMS staining of the retina to quantify RGC counts demonstrated significant structural protection of RGCs (Figure 6, E and F). Together, these results show that LNP-ABE-A7 effectively lowers IOP, prevents RGC functional and structural loss, and reduces MYOC aggregates, thereby rescuing glaucoma in a mouse model of *MYOC*-associated POAG.

### LNP-ABE-A7 exhibits excellent ocular safety without any significant off-target effects in vivo.

**Figure 7:**
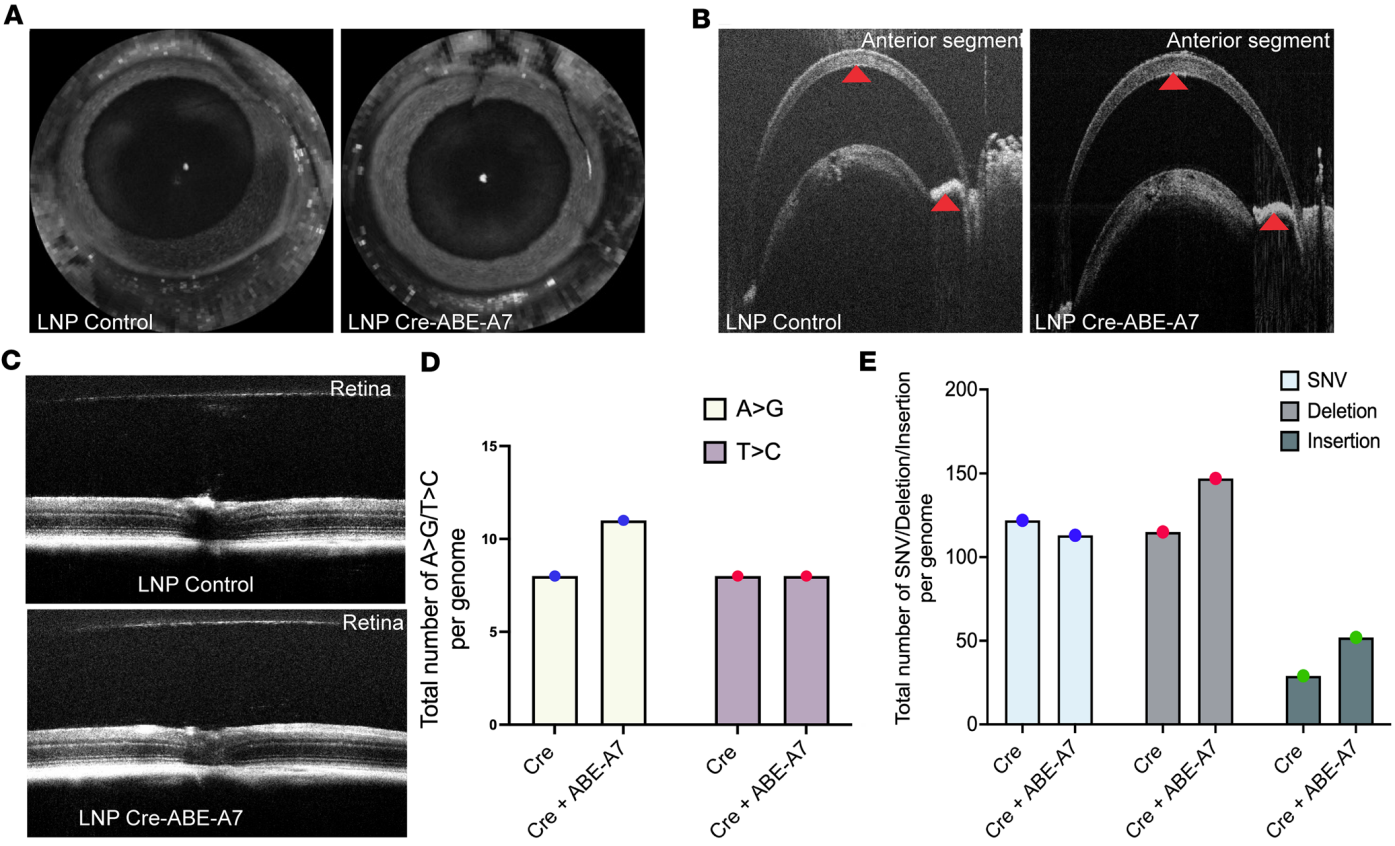
LNP-ABE-A7 shows no ocular toxicity or off-target editing effects in *Tg.Cre-MYOC^Y437H^* mice. Adult *Tg.CreMYOC^Y437H^* mice were injected with LNP-ABE-A7, and ocular structures were analyzed via optical coherence tomography (OCT) imaging of the anterior and posterior segments. (**A**–**C**) OCT imaging of the anterior (**A** and **B**) and posterior (**C**) segments revealed no structural abnormalities in eyes treated with LNP-ABE-A7 compared to controls. Red arrowheads in **B** indicate the anterior chamber angle, and white arrowheads indicate corneal thickness. (**D** and **E**) Whole-genome sequencing (WGS) of genomic DNA from TM tissue demonstrated that LNP-ABE-A7 did not induce significant off-target effects. (**D**) The number of A-to-G or T-to-C substitutions was comparable between ABE-A7–treated and Cre-only control samples. (**E**) Similarly, the total number of SNVs, deletions, and insertions per genome was unchanged between groups. *n* = 6 combined *Tg.CreMYOC^Y437H^* mice. SNV, single-nucleotide variation.

A critical step in translating gene-editing technology into humans is to ensure its safety. We first explored whether LNP-ABE-A7 exhibits any ocular toxicity using optical coherence tomography (OCT) in vivo. Adult *Tg.CreMYOC^Y437H^* mice injected with LNP-ABE-A7 were analyzed by live OCT imaging 10 weeks after ABE treatment (Figure 7, A–C). Anterior segment OCT images clearly show no evidence of ocular inflammation in the eyes of LNP-ABE-A7–treated mice compared with controls (Figure 7A). The iridocorneal angle is open, and the corneal thickness is similar between LNP-ABE-A7– and LNP-buffer–injected eyes (Figure 7B). Posterior segment OCT also revealed no changes in retinal layers of LNP-ABE-A7–injected eyes (Figure 7C and Supplemental Figure 6). These data establish the ocular safety of LNP-ABE-A7. Previously, we observed significant off-target effects when Cas9 was delivered via LV particles to mouse TM (19). Here, we examined whether LNP–ABE mRNA exhibits any off-target effects using whole-genome sequencing (WGS) of genomic DNA obtained from the TM of LNP-Cre– or LNP-Cre– plus LNP-ABE-A7–treated *Tg.Cre-MYOC^Y437H^* mice. Note that NGS data demonstrated 17% on-target editing efficiency on these samples. The top candidate off-target sites revealed no meaningful differences in A-to-G or T-to-C substitutions between the 2 groups (Figure 7D). The total number of A-to-G and T-to-C substitutions across the genome was similar in both untreated and treated Cre-injected *Tg.CreMYOC^Y437H^* mice, indicating that these substitutions likely represent background noise rather than true off-target activity. Annotation of single-nucleotide variants (SNVs), deletions, and insertions at candidate sites further confirmed the absence of detectable off-target editing in LNP-ABE-A7–treated samples (Figure 7E). Together, these findings demonstrate that LNP-ABE-A7 achieves efficient on-target editing without inducing significant off-target effects in vivo.

## Discussion

Mutations in *MYOC* are the major cause of POAG, especially in children. *MYOC*-associated glaucoma in children usually presents clinically with elevated IOP, progresses rapidly to vision loss, and is often less responsive to existing treatments since these treatments do not target the underlying cause (6). Targeting *MYOC* via genome editing holds great promise in developing precision therapeutics for glaucoma (18, 19). While WT *MYOC* is not required for IOP regulation, mutations in the gene cause a deleterious gain of function, leading to TM cell death and IOP elevation. Therefore, knocking out *MYOC* via genome editing provides an ideal strategy to target over 100 pathological *MYOC* mutations and provide a permanent “cure” for *MYOC*-associated glaucoma. Previously, we demonstrated that viral vector–mediated delivery of Cas9 successfully edited *MYOC* and rescued glaucoma in mice (18, 19). However, a key challenge in advancing genome-editing therapies for glaucoma remains the development of a robust and selective tissue-specific delivery system, along with precise and efficient gene-editing tools that minimize off-target effects (19). The findings from the current study offer a tremendous advancement toward developing gene editing for the treatment of glaucoma by addressing these key concerns, including the demonstration of highly specific delivery of Cas9 mRNA to the TM using LNPs, and utilization of ABEs to minimize off-target effects with enhanced editing efficiency. Using the LNP-mediated delivery of ABEs, we have successfully edited the *MYOC* gene with high efficiency. In addition, the use of ABEs minimized off-target effects and enhanced on-target editing. Importantly, our strategy of changing the initiation codon from ATG to GTG effectively silences *MYOC* expression, thereby addressing all pathogenic mutations associated with *MYOC*-linked POAG. With over 100 different *MYOC* mutations implicated in glaucoma (3, 7), this gene-knockout approach provides a universal therapeutic strategy to target all pathogenic variants.

Over the past several years, LNP-mediated delivery of base editors has emerged as a promising strategy for precise and efficient gene editing. LNP-based tools have been applied successfully to target the liver, lungs, blood cells, and eyes (30–38). The eye is an especially advantageous system for developing and optimizing gene-editing therapies due to its accessibility, the ability to deliver therapeutics locally, the availability of advanced functional and imaging modalities, and the feasibility of directly measuring treatment outcomes.

Although gene editing holds great promise for targeting pathological genes in glaucoma, the lack of TM specificity with current viral vectors has limited its translational potential. Our study demonstrates that LNPs exhibit highly specific tropism for the TM. Compared with traditional viral vectors, which transduce TM cells sporadically, LNPs transduced nearly the entire TM, as evidenced by robust and widespread mCherry expression following LNP–mCherry mRNA delivery. Moreover, LNP–Cre mRNA produced uniform and strong Cre activity throughout the TM. Notably, a direct comparison revealed that LNPs outperformed both LV and AAV in delivering reporter genes to the TM, achieving markedly higher efficiency and selectivity. In addition, while LV induced ocular inflammation as reflected by increased TNF-α and GFAP expression, LNPs exhibited no evidence of inflammation, highlighting their superior safety profile.

This conclusion was further supported by the development of glaucoma in *Tg.Cre-MYOC^Y437H^* mice after LNP-Cre injection. Cre-injected *Tg.Cre-MYOC^Y437H^* mice exhibited robust and sustained IOP elevation without any signs of ocular inflammation. Finally, a single injection of LNP-ABE demonstrated 17% editing of *MYOC*, which was sufficient to decrease the accumulation of mutant MYOC and ablate the symptoms in a mouse model of *MYOC*-associated glaucoma. Consistent with this observation, a few recent studies have shown that LNPs or lipoplex complexes can deliver mRNA to the iridocorneal angle or TM tissue in mice (39, 40). We have recently demonstrated (40) that a single IC injection of lipoplex encapsulating mRNA exhibited strong tropism to the TM and lipoplex encapsulating Cas9 mRNA with sgRNA targeting mutant *MYOC* rescued *Tg.CreMYOC^Y437H^* mice without any ocular toxicity. Together, these studies highlight LNP-mRNA as an attractive platform to deliver therapeutics to the TM.

Although we estimated a modest 17% editing efficiency in FACS-isolated TM cells, this value is likely an underestimate due to several limitations. First, isolating pure TM cells is challenging due to their anatomical location, and contamination with scleral or ciliary body cells, which LNP-ABE does not target, likely reduced the apparent editing efficiency. Second, sorting was based on DsRed expression covalently linked to mutant MYOC, which is expected to decrease following successful editing; although FACS was performed 1 week after LNP-ABE injection to minimize this effect, some edited cells were likely excluded. Third, Western blot analysis revealed a 46% reduction in DsRed-MYOC protein across the entire TM, consistent with a greater functional impact of editing than that reflected in the NGS data. Importantly, true editing efficiency is reflected by the significant reduction in MYOC protein and its aggregates, since we leveraged DsRed-tagged mutant MYOC as a direct readout in the iridocorneal angle tissue. Because DsRed expression is restricted to the TM following Cre induction, Western blotting or immunostaining for DsRed provides a selective and reliable measure of editing efficiency in this tissue. These findings are further supported by the complete rescue of glaucomatous phenotypes. Notably, we observed a significant reduction in protein aggregates. Although gene editing does not directly eliminate aggregates, it reduces total mutant protein load, thereby allowing endogenous degradation mechanisms such as the proteasome and autophagy to clear preexisting aggregates.

Since the mouse eye can hold approximately 3–4 μL of aqueous humor, injecting it with more than 3–4 μL of LNP-ABE is not feasible. However, more than 100 μL of LNP-ABE can be injected routinely into the human eye, which may enhance editing efficiency. Moreover, multiple intraocular injections can further increase the editing efficiency of LNP-ABE. It is interesting to note that gene editing of ocular-hypertensive mice not only decreased IOP significantly but also prevented glaucomatous neurodegeneration. This observation suggests that gene-editing treatment during the early stage of pathology may completely reverse the disease and provide a permanent cure for pediatric glaucoma patients affected by these mutations.

A major limitation of clinical applications of genome editing has been the lack of a robust and selective tissue-specific delivery system and potential off-target effects. An ideal delivery platform for genome-editing tools should be tissue-specific and exert no off-target effects. Moreover, Cas9 nuclease should be expressed transiently and rapidly degraded after editing to reduce its action at less favored binding sites on the genome and transcriptome (35). The present study has addressed these concerns. The LNPs exhibited robust and selective TM tropism in vivo and demonstrated robust editing efficiency in the entire TM. Recent advancements in mRNA technology have made it possible to deliver genome editors in vivo in certain cell types. These mRNA platforms offer several advantages, including low immunogenicity, high tropism to the TM based on its phagocytic nature, and fewer off-target effects compared with the traditional viral vectors (35, 36, 41). LNP-mediated delivery of ABE mRNA produced Cas9 within 24 hours and then was rapidly degraded within 48 hours. In addition, base editors have been shown to exhibit higher editing efficiency compared with traditional Cas9, without any off-target effects (27). NGS analysis demonstrated no bystander effect of using A7 sgRNA in vivo, confirming high specificity with robust on-target editing using base editors.

Inhibition of phagocytosis with cytochalasin D significantly blocked LNP uptake by primary TM cells, supporting phagocytosis as the likely mechanism. TM cells are highly phagocytic and continuously clear cellular debris from the aqueous humor (42–45), a property that may naturally facilitate LNP internalization. Similarly, retinal pigmented epithelium, which is composed of another phagocytic cell type, has been shown to exhibit strong tropism for LNPs (36). Thus, a highly active endocytic pathway may underlie the rapid and selective uptake of LNPs by TM cells. Beyond uptake, LNPs are well suited for mRNA delivery because they both protect the cargo and promote cellular entry. Ionizable lipids, in particular, shield mRNA from endosomal degradation. Modifying lipid composition in future studies may further optimize LNP formulations to enhance editing efficiency.

Unlike currently used viral vectors, the LNP-ABE formulation in our study induced no ocular inflammation, as confirmed by OCT imaging of both anterior and posterior segments, an essential requirement for clinical translation. In our previous work, LV-mediated Cas9 delivery resulted in significant genomic off-target effects (19). In contrast, WGS in the present study revealed no detectable off-target activity, while demonstrating robust on-target base editing after LNP-ABE treatment. Together, these results highlight the safety of LNP-based genome editing in a preclinical model.

Although the deaminase activity of base editors can, in some cases, alter RNA (24), such effects are expected to be minimal with LNP-mediated delivery because the editor complex is short-lived compared with viral systems. Consistent with this, NGS detected no significant bystander activity in LNP-ABE–treated mice. Moreover, potential bystander edits are less relevant in this context, as our strategy is designed to disrupt gene expression rather than preserve the exact coding sequence. While this study focuses on adenine base editing, the LNP platform is highly versatile and can be adapted to deliver cytidine base editors, prime editors, or traditional Cas9 nucleases (46). Beyond gene editing, LNPs can also be engineered to deliver therapeutic proteins to the TM, enabling intervention in additional pathogenic pathways.

In summary, we demonstrate that LNP-mediated mRNA delivery efficiently and selectively targets the mouse TM without ocular toxicity. Using LNP–Cre mRNA, we induced mutant *MYOC* expression in an inducible mouse model carrying the human *Y437H-MYOC* mutation, which developed a robust glaucoma phenotype closely resembling human POAG. Importantly, LNP-ABE-A7–mediated gene editing successfully disrupted mutant *MYOC*, reduced its accumulation, and rescued glaucomatous pathology in vivo. Together, these findings establish proof of concept for base editing as a variant-independent therapeutic strategy in the TM, providing a long-term, efficient, and potentially curative approach for *MYOC*-associated glaucoma.

## Methods

### Sex as a biological variable.

The study included animals of both sexes, but sex-based differences were not specifically examined or factored into the analysis.

### Animal husbandry.

The animals were housed and bred under controlled 12-hour light/dark cycles, provided with standard chow ad libitum, and kept in cages with dry bedding. Two- to 3-month-old C57BL/6J mice (both male and female) were obtained from The Jackson Laboratory. mTmG mice (stock 007576) were obtained from The Jackson Laboratory, and 2- to 3-month-old mice were used in the present study, as described previously (47). Our laboratory has recently generated Cre-inducible transgenic mice expressing human mutant Y437H-MYOC (*Tg.CreMYOC^Y437H^* mice) on a pure C57BL/6J background (29). Six-month-old male and female *Tg.CreMYOCY^437H^* mice were utilized in the current study. Euthanasia was performed using carbon dioxide inhalation followed by cervical dislocation in accordance with an approved animal protocol.

### Antibodies and reagents.

The following antibodies and reagents were used in the current study. DsRed-MYOC was detected using an RFP-conjugated antibody (catalog 600-401-379, Rockland). Other primary antibodies utilized were against KDEL (catalog ab12223, Abcam), RBPMS (catalog 118619, Gene Tex), GAPDH (catalog 3683, Cell Signaling Technology), TUJ1 (catalog GTX130245, Genetex), Cas9 (catalog 84430, BioLegend), GFAP (catalog ab4674, Abcam), CHOP (catalog NBP2-13172, Novus), and TNF-α (catalog ab1793, Abcam). Reagents used were goat serum (EMD Millipore, S26-LITER), Triton X-100 (Sigma-Aldrich, T8787), Tween 20 (Sigma-Aldrich, P9416), RIPA buffer (Thermo Fisher Scientific), PBS (Genesee Scientific, 25-508), proparacaine hydrochloride (SANDOZ), paraformaldehyde (PFA) 16% Aqueous Solution EM Grade (EMS, 15700), PVDF membranes (MilliporeSigma), DAPI mounting solution (Vectashield, Cole Parmer), collagenase (Thermo Fisher Scientific, 17104019), MOU-modified mCherry mRNA (TriLink BioTechnologies, L-7203-1000), MOU-modified Cre mRNA (TriLink BioTechnologies, L-7211-1000), DMEM/F-12 (Thermo Fisher Scientific, 10565018), primers and sgRNAs (Integrated DNA Technologies), cytochalasin D (Thermo Fisher Scientific, PHZ1063), AAV-Cre (Signagen, SL116032), LV-GFP (Vector Builder, Inc, LVMP-VB160109-10005), and Papain Dissociation System (Worthington Biochemical Corporation).

### Synthesis of mRNA and sgRNA.

The MOU-modified mRNAs for mCherry and Cre were obtained from TriLink BioTechnologies. ABE mRNA (ABE8e-SpCas9-NG) and ABE MOU-mRNA were commercially synthesized (Azenta Life Sciences) by in vitro transcription from a template plasmid, as previously described (35). Briefly, these genes were synthesized and cloned into the pUC-GW-Kan plasmid under a T7 promoter. In vitro transcription of both modified and unmodified mRNA and its quality control was performed by Azenta Life Sciences. The designed MYOC sgRNAs, A7 and A9, were synthesized and acquired from Integrated DNA Technologies.

### LNP formulation.

The mRNA for mCherry or Cre alone or ABE mRNA and sgRNAs together were encapsulated in LNPs. The LNP formulation included the ionizable cationic lipid ALC0315 (Avanti Polar Lipids, 890900), a key lipid component of Pfizer’s approved COVID-19 vaccine, combined with helper lipids DSPC (Avanti Polar Lipids, 850365), cholesterol (Avanti Polar Lipids, 700100), and PEG2000-DMG (Avanti Polar Lipids, 880151) at a molar ratio of 50:10:38.5:1.5, similar to those used in Pfizer and Moderna’s COVID-19 vaccines (48–51). The chemical structures of these lipids are provided in the Supplemental Information S1A. In brief, the ABE mRNA and sgRNA targeting MYOC were coencapsulated in LNPs at a 1:1 weight ratio, as described previously (41, 52). The lipids were dissolved in ethanol and rapidly combined with mRNA/sgRNA in 100 mM sodium acetate buffer at pH 4 in a volumetric ratio of 1:3 (ethanol/aqueous). A molar ratio of 6 was used for ionizable lipid nitrogen/nucleic acid phosphate (N:P) (commonly known as the charge ratio of cationic lipid to the negatively charged RNA). The combination was performed by microfluidic mixing using Precision NanoSystem’s Ignite, or rapid mixing via T-junction using a dual syringe pump. The ionizable lipid became protonated at low pH and bound electrostatically to the anionic phosphate backbone of the mRNA and sgRNA, driving the vesicle formation and RNA encapsulation. The pH was then neutralized by dialysis with more than 100 volumes of 20 mM Tris/4.3 mM acetate/10% sucrose buffer pH 7.4 using a Pur-A-Lyzer dialysis device with a molecular weight cutoff (MWCO) of 12 kDa (Sigma-Aldrich, PURX12015) for at least 5 hours to form neutral LNPs. Ethanol was simultaneously removed during dialysis. The final LNPs were concentrated using a centrifugal ultrafilter (Amicon Ultra-4, UFC801024; MWCO 10 kDa) and then sterile filtered. LNP control formulations contained the same lipid composition as the LNP-mRNAs but lacked mRNA, serving as vehicle-only controls.

### NGS and data analysis.

Genomic DNA was isolated from the cultured TM cells and the TM of *Tg.CreMYOC^Y437H^* mice after gene editing, using a DNA isolation kit (Qiagen, 69504) per the manufacturer’s protocol. Of the isolated genomic DNA, 1–2 μL was used as input for the PCR reactions. Genomic loci were PCR amplified using Phusion Plus polymerase (Thermo Fisher Scientific, F631S). Two hundred fifty-two–base pair regions of *MYOC* were PCR amplified using MYOC-NGS-FW (CCGGGGGATCCCCATCAA) and *MYOC*-NGS-REV (CTGGACAGCTGGCATCTCA) primers, along with NGS partial adapters, according to the manufacturer’s instructions (Azenta Life Sciences). The amplicons were then purified using a PCR purification kit (Invitrogen, K310001) according to the manufacturer’s protocol. Azenta Life Sciences performed the amplicon sequencing and its analysis. The editing efficiency of LNP-encapsulated ABE mRNA was quantified by comparing the reference sequence to the percentage of edited reads. Data analysis was performed using GraphPad Prism.

### Particle size distribution analysis.

The particle size distribution was assessed with a Malvern Zetasizer Advance Nano (Malvern Panalytical). A 20 μL sample of newly dialyzed LNP was mixed with Tris/acetate/saline buffer to reach a total volume of 200 μL. This mixture was then analyzed in triplicate to determine the particle size distribution. The resulting data were evaluated using the device’s software, which provided the average particle diameter and the polydispersity index (PdI).

### Cryoelectron microscopic imaging of LNPs.

LNP samples were first concentrated 3- to 4-fold using an Amicon Ultra 0.5 device featuring a 10 kDa MWCO. A 2.5-μL portion of the LNP solution was then placed onto a Quantifoil 200-mesh grid, which had been coated with a thin carbon layer (Ted Pella). The grids were gently blotted with filter paper for 2 seconds before being rapidly submerged in liquid ethane with a manual plunger. Imaging was performed using an FEI Tecnai TF20 high-resolution transmission electron microscope, fitted with a K2 direct detection camera, operating at 200 kV.

### LNP-mCherry phagocytosis assay in human primary TM cells.

Human primary TM cells (*n* = 3 donor cell strains) were cultured under standard conditions until approximately 80% confluence, as described previously (53–55). Cells were pretreated with 1 μM cytochalasin D (phagocytosis inhibitor) (28) for 24 hours, followed by incubation with LNP-mCherry (2 μg/mL) for 48 hours. After incubation, cells were washed with PBS to remove unbound particles and fixed with 4% PFA. Nuclei were counterstained with DAPI, and mCherry uptake was assessed using confocal microscopy. Phagocytosis was quantified by measuring mCherry fluorescence intensity per TM cell using ImageJ software (NIH).

### Evaluation of MYOC editing in mouse outflow cells.

Outflow cells were isolated from *Tg-CreMYOC^Y437H^* mice immediately after enucleation. Eyes were placed in ice-cold PBS, and the TM ring was carefully dissected from the anterior segment with meticulous removal of surrounding tissues. Outflow cells were cultured and utilized for *MYOC* editing in vitro. For evaluation of mutant *MYOC* gene editing, cultured mouse outflow cells were treated with either LNP-Cre alone (to induce mutant *MYOC*; 2 μg/mL) or a combination of LNP-Cre and LNP-ABE-A7 (2 μg/mL each) for 72 hours. After treatment, cells were fixed with 4% PFA and immunostained for ER stress markers.

### IC injections of LNPs.

LNPs encapsulating mRNA were injected IC using a slow infusion pump. Mice were anesthetized via an intraperitoneal injection of ketamine (100 mg/kg) and xylazine (10 mg/kg). Prior to IC injection, mouse eyes were anesthetized by topical administration of proparacaine HCl drops (0.5%; Akorn Inc.). Mouse eyes were treated topically with 1% cyclopentolate (Mydriacyl, Alcon Laboratories) to dilate the pupils. The glass micropipette loaded onto a microdialysis infusion pump (SP101I Syringe Pump; WPI) was used to deliver LNPs at a flow rate of 0.6 μL/min over the course of 7–8 minutes (total volume delivered, 4 μL). A drop of filtered saline was also applied through this procedure to prevent corneal drying. This delivery rate approximates the normal rate of aqueous humor production and therefore does not acutely elevate IOP. Prior to injection, a small amount of aqueous humor was allowed to egress to further minimize transient pressure elevation and prevent reflux of the injected material. No corneal edema, anterior segment deformation, or other ocular abnormalities were observed during or after the procedure. To assess mCherry tropism in mouse TM, IC injection of LNP-mCherry (1.48 μg/eye) or LNP-buffer (control) was administered by slow perfusion. Animals were sacrificed 2 days after injection, and the eyes were fixed with 4% PFA. The fluorescence of the mCherry protein was examined via confocal microscopy in flat mounts and cross sections of the anterior segments. LNP–Cre mRNA (1.46 μg/eye) was injected IC in mTmG mice as described above. To induce mutant MYOC in *Tg.CreMYOC^Y437H^* mice, a slow perfusion of LNP-Cre (1.47 μg/eye) or LNP-buffer via the IC route was performed. For *MYOC* gene editing in *Tg.CreMYOC^Y437H^* mice, 6-month-old *Tg.CreMYOC^Y437H^* mice were injected IC with LNP-ABE-A7 (1.52 μg/eye). A slow perfusion of 4 μL LNPs was performed as described above. One week after injection, another IC injection of LNP-Cre was performed to induce mutant *MYOC*. Two weeks after the injection, the mouse iridocorneal angle tissue was isolated and digested with papain. DsRed-positive TM cells were sorted using FACS. Genomic DNA was isolated from sorted TM cells and analyzed by NGS. To examine the effect of *MYOC* editing on glaucoma phenotypes, 6-month-old *Tg.CreMYOC^Y437H^* mice were injected IC with LNP-Cre to induce mutant *MYOC*. IOP was monitored weekly to track IOP elevation in *Tg.CreMYOC^Y437H^* mice. Five weeks after injection, ocular-hypertensive mice were divided into 2 groups: 1 group received LNP-ABE-A7, and the other group received LNP-buffer. IOP was monitored weekly in all 3 groups for up to 10 weeks. PERG and RGC analyses were performed at 15 weeks. All data collection was conducted in a masked manner.

### IC injections of viral vectors.

IC injection of LV-GFP was performed as in a previous study (19), and parts of these experiments were reused in the present study for direct comparison with LNP-mediated delivery. Briefly, sections were cut from blocks frozen in Optimal Cutting Temperature (O.C.T.) compound (Tissue-Tek) and mounted with DAPI. For Cre activity, mTmG reporter mice were injected IC with AAV2-Cre (3.14 × 10^^13^^ VG/mL; 2 μL) or LV-Cre (1.41 × 10^^9^^ TU/mL; 2 μL).

### Western blot analysis.

TM cells were lysed in RIPA buffer (Thermo Fisher Scientific) as described previously (54, 56). Approximately 20 μg of total protein was loaded per lane and separated in 4%–12% NuPAGE Bis-Tris gradient gels (Life Technologies). Proteins were then transferred onto PVDF membranes. Membranes were blocked with 10% nonfat dried milk for 1 hour, followed by overnight incubation at 4°C with specific primary antibodies, on a rotating shaker. After 3 PBST washes, membranes were incubated for 90 minutes with HRP-conjugated secondary antibodies. Protein detection was performed using the LICOR Odyssey Fc imaging system with ECL detection reagents (Super Signal West Femto, Life Technologies). Blots were probed with anti-GAPDH antibody to confirm equal protein loading.

### Anterior segment flat mount.

To visualize DsRed-MYOC levels in the TM region, anterior segment flat mounts were prepared. Eyes from *Tg.CreMYOC^Y437H^* mice injected with LNP-Cre and LNP-ABE-A7 were enucleated, fixed in 4% PFA, and the retinas dissected into the anterior and posterior segments. The anterior segments were placed on a slide, cut into 4 quadrants for optimal flattening, and mounted with a DAPI-containing solution. Images were captured using a Keyence microscope (Itasca).

### Immunostaining.

Eyes were enucleated and fixed in 4% PFA for 3 hours before embedding them in paraffin or O.C.T. compound, as described previously (57). Five- or 10-micron sections were utilized for immunostaining. The slides were washed with PBS. Deparaffinized or O.C.T. compound–treated slides were incubated with a blocking buffer containing 10% goat serum and 0.5% Triton-X-100 in PBS for 2 hours. Primary antibodies were diluted in a blocking buffer and incubated with the slides overnight. After 3 washes with PBS, the slides were incubated with Alexa Fluor–conjugated secondary antibodies (Life Technologies) for 2 hours. The sections were washed thrice with PBS and mounted with a DAPI-containing mounting solution. Images were captured using a Leica SP8 confocal microscope. For all percentage measurements (e.g., Figures 2 and 5), the total TM area was determined by first delineating the anatomical boundaries of the TM region within the iridocorneal angle. Fluorescence intensity of GFP/DsRed was then measured specifically within this defined TM region, and values were normalized to the total TM area to yield percentage targeting or protein expression.

### IOP measurements.

IOP was measured weekly using a TonoLab rebound tonometer (Colonial Medical Supply); the mice were maintained under gas anesthesia using isoflurane (2.5%) and oxygen (0.8 L/min), as described previously (58, 59). All IOP measurements were conducted between 9 am and 2 pm and replicated independently in a masked manner. The final IOP value was determined by averaging 6 individual measurements.

### PERG.

RGC function was determined using a binocular snout PERG system (JORVEC Corp.), as previously described (60). Anesthetized mice were positioned on a temperature-controlled metal base, 10 cm from LED monitors, and their body temperature was maintained at 37°C using a rectal probe. A small amount of Hypromellose eye drops was applied topically to prevent corneal dryness during the recording. The PERG signals were recorded simultaneously from both eyes using subcutaneous electrodes placed at the snout (active), the back of the head (reference), and the tail (ground). The signals were generated in response to contrast reversals of gratings displayed on 2 LED screens operating at slightly different frequencies.

### Whole-mount retina staining for RBPMS.

The total number of RGCs was determined after staining the whole-mount retina with an anti-RBPMS antibody, as previously described (60). Enucleated eyes were fixed in 4% PFA for 12 hours at 4°C. After rinsing with PBS, the posterior segment was carefully isolated, and the retinas were peeled from the posterior eye cup. The isolated retinas were incubated in a blocking buffer containing PBS, 10% goat serum, and 0.2% Triton X-100 for 12 hours at 4°C. The retinas were incubated with the anti-RBPMS antibody for 3 days at 4°C. After a 2-hour PBS wash, the retinas were incubated with a goat anti-rabbit–Alexa Fluor 568 secondary antibody (1:500; Invitrogen) for 2 hours at room temperature. The retinas were washed 3 times with PBS and then mounted using a DAPI-containing mounting medium. To quantify RGCs, at least 16 nonoverlapping images of the entire retina were taken at ×200 magnification using a Keyence fluorescence microscope (Itasca). RBPMS-positive cells were counted using ImageJ software.

### OCT imaging.

Mice were anesthetized using an intraperitoneal injection of a ketamine/xylazine mixture (100 mg/kg and 10 mg/kg, respectively). Mouse eyes were treated topically with 1% cyclopentolate (Mydriacyl, Alcon Laboratories) to dilate the pupils. The animals were properly positioned, and OCT scans were automatically acquired using the Leica OCT system with a quality index above 28. Retinal structure was assessed using a linear scan, while anterior segment images were captured by adjusting the focus and increasing the distance from the cornea. Augmented software was used to measure retinal thickness and corneal parameters.

### WGS.

WGS was performed to assess potential off-target effects of ABE-A7 in *Tg.CreMYOC^Y437H^* mice. Genomic DNA was isolated from TM tissue using the DNeasy Blood & Tissue Kit (Qiagen, 69504). Libraries were prepared and sequenced by Genewiz/Azenta (https://www.genewiz.com/public/services/next-generation-sequencing/whole-genome-sequencing), who also conducted the primary analysis. Off-target assessment focused on A-to-G and T-to-C edits, as well as SNVs, insertions, and deletions. Raw reads were trimmed with Trimmomatic 0.39 (https://trimmomatic.com/) and aligned to the *Mus musculus* GRCm38 reference genome using Sentieon 2023.08.02 (https://www.sentieon.com/publications/https:/, /www.sentieon.com/products/). Alignments were sorted, and PCR/optical duplicates were marked. SNVs and INDELs were called using DNAScope (https://github.com/Sentieon/sentieon-models.git). Resulting VCFs were normalized using bcftools 1.13 (left-alignment and multiallelic splitting) (https://www.htslib.org/doc/1.1/bcftools.html). Variant consequences were annotated using Ensembl VEP 104 (https://www.ensembl.org/index.html?redirect=no). Candidate variants were compared between LNP-Cre–treated and LNP-Cre– plus LNP-ABE-A7–treated TM DNA to identify potential ABE-A7–associated off-target edits, as described previously (61, 62).

### Statistics.

Statistical analyses were conducted using Prism 9.0 software (GraphPad). Results are expressed as mean ± SEM, with *n* representing the number of eyes analyzed. A *P* value of less than 0.05 was considered statistically significant. Group comparisons were performed using a 2-tailed Student’s *t* test for 2-group analyses. For multiple treatment comparisons, a 2-way ANOVA was used, followed by post hoc analyses. All data analyses were conducted in a masked manner.

### Study approval.

Animal studies were conducted in accordance with the guidelines outlined in the Association for Research in Vision and Ophthalmology (ARVO) Statement for the Use of Animals in Ophthalmic and Vision Research. The Institutional Animal Care and Use Committee (IACUC) and Biosafety Committees at the University of California, Irvine approved all experimental protocols.

### Data availability.

The numerical data underlying all figures and reported means are provided in the accompanying Supporting Data Values file. Raw WGS data are available from the NCBI Sequence Read Archive (SRA) under BioProject accession PRJNA1372897 (https://www.ncbi.nlm.nih.gov/sra/PRJNA1372897). Additional datasets generated and analyzed during the current study are available from the corresponding author upon reasonable request.

## Authors contributions

GZ, KP, and BRK conceptualized the research studies, and GZ and BRK designed them. BRK and LL performed experiments and analyzed data. JF and PF prepared lipid nanoparticles. PG and SD assisted in some experiments. BRK and GZ wrote the manuscript, and KP, JF, and PF assisted with editing. All authors discussed the results and implications and commented on the manuscript at all stages.

## Funding support

This work is the result of NIH funding, in whole or in part, and is subject to the NIH Public Access Policy. Through acceptance of this federal funding, the NIH has been given a right to make the work publicly available in PubMed Central.

- NIH grants EY034333 (to GZ), EY026177 (to GZ), EY030366 (to GSZ), R01EY036994 (to KP), R01EY034501 (to KP), and EY034238.
- Alcon Research Foundation (to GZ).
- Glaucoma Research Foundation’s Shaffer Grant (to BRK).
- Research to Prevent Blindness (to GZ).
- Research to Prevent Blindness (unrestricted grant to the Gavin Herbert Eye Institute at the University of California, Irvine).

## Supplementary Material

Supplemental data

Unedited blot and gel images

Supporting data values

## Version 1 02/09/2026

Electronic publication
