# Supplementary material for: Lipid-nanoparticle-mediated base editing of the trabecular meshwork rescues glaucoma in vivo
Source: JCI Insight. 2026 Feb 9;11(3):e195593. doi: 10.1172/jci.insight.195593 (PMC12892903; doi:10.1172/jci.insight.195593)
Supplement: Supplemental data [file jciinsight-11-195593-s092.pdf]

Supplemental Information
Kaipa et al.

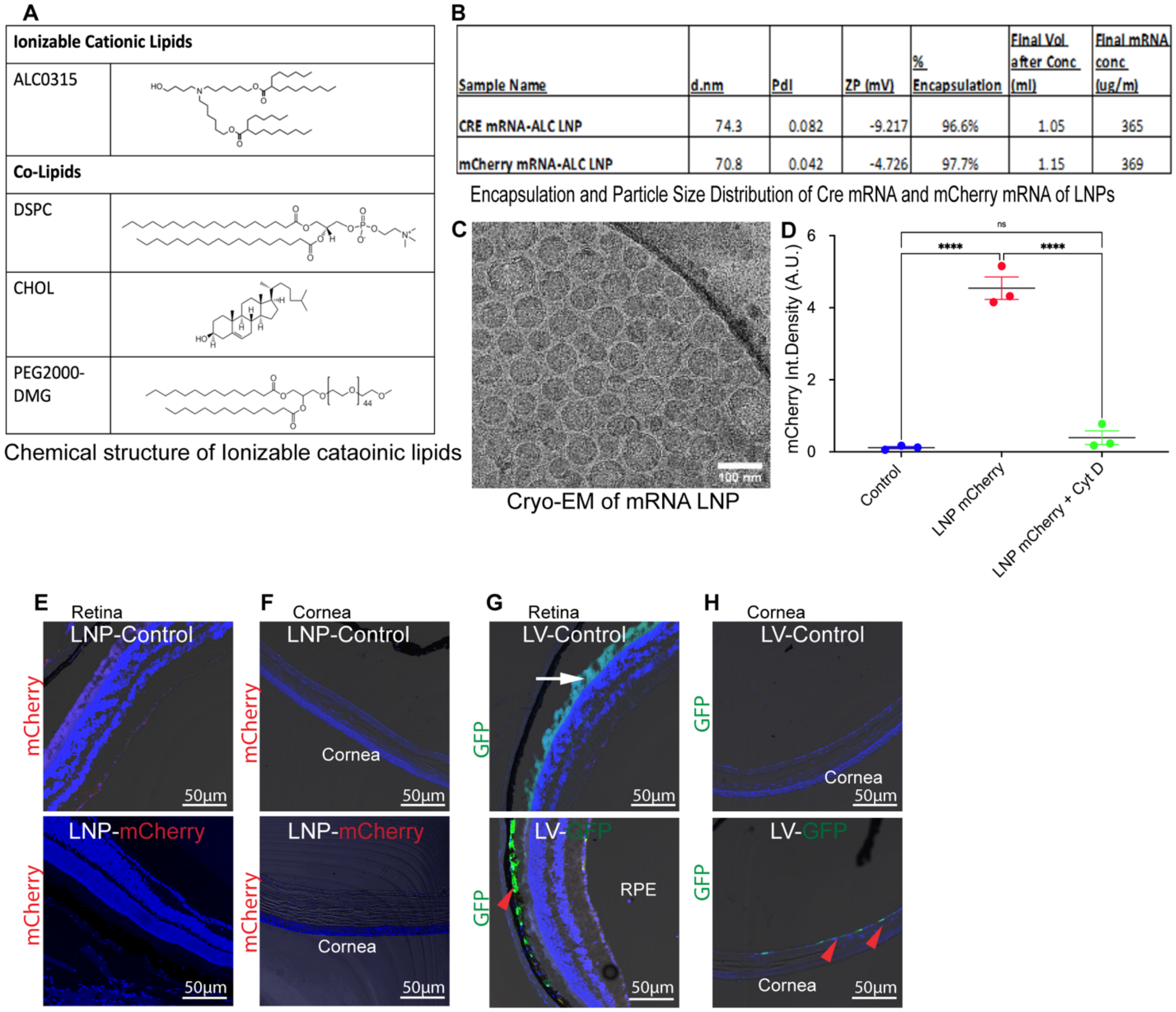

Sample Name	d.nm	PdI	ZP (mV)	% Encapsulation	Final Vol after Conc (ml)	Final mRNA conc (ug/m)
CRE mRNA-ALC LNP	74.3	0.082	-9.217	96.6%	1.05	365
mCherry mRNA-ALC LNP	70.8	0.042	-4.726	97.7%	1.15	369

Supplementary Figure S1. LNPs exhibit efficient encapsulation, stable physicochemical properties, and selective tropism for TM cells.

(A) Schematic showing the chemical structures of the ionizable cationic lipid ALC-0315 and co-lipids (DSPC, cholesterol, PEG2000-DMG) used to formulate lipid nanoparticles (LNPs).

(B) Physicochemical characterization demonstrated that both LNP-mCherry and LNP-Cre exhibited >96% encapsulation efficiency with uniform particle size (~70–75 nm), narrow polydispersity index, and stable zeta potential, confirming robust and reproducible nanoparticle formulations.

(C) Representative cryo-EM imaging revealed spherical, uniformly sized mRNA-loaded LNPs with consistent encapsulation of nucleic acids, further validating their structural stability.

(D) Quantitative analysis of mCherry integrated density in primary TM cells (n=3 donor cell strains) confirmed highly efficient uptake of LNP-mCherry, achieving near-complete transfection (~100%). Treatment with cytochalasin D, a phagocytosis inhibitor, significantly reduced mCherry uptake, establishing phagocytosis as the major mechanism of LNP entry.

(E–F) In vivo intracameral delivery of LNP-mCherry in C57 mice demonstrated strong, selective mCherry expression confined to the TM, with no detectable expression in the retina, retinal pigment epithelium (RPE), or corneal endothelium.

(G–H) By contrast, LV delivery resulted in ectopic GFP expression in the RPE and corneal endothelium, highlighting the superior tissue specificity of LNP-mediated mRNA delivery. Arrowheads point to RPE (G) or corneal endothelial cells (H).

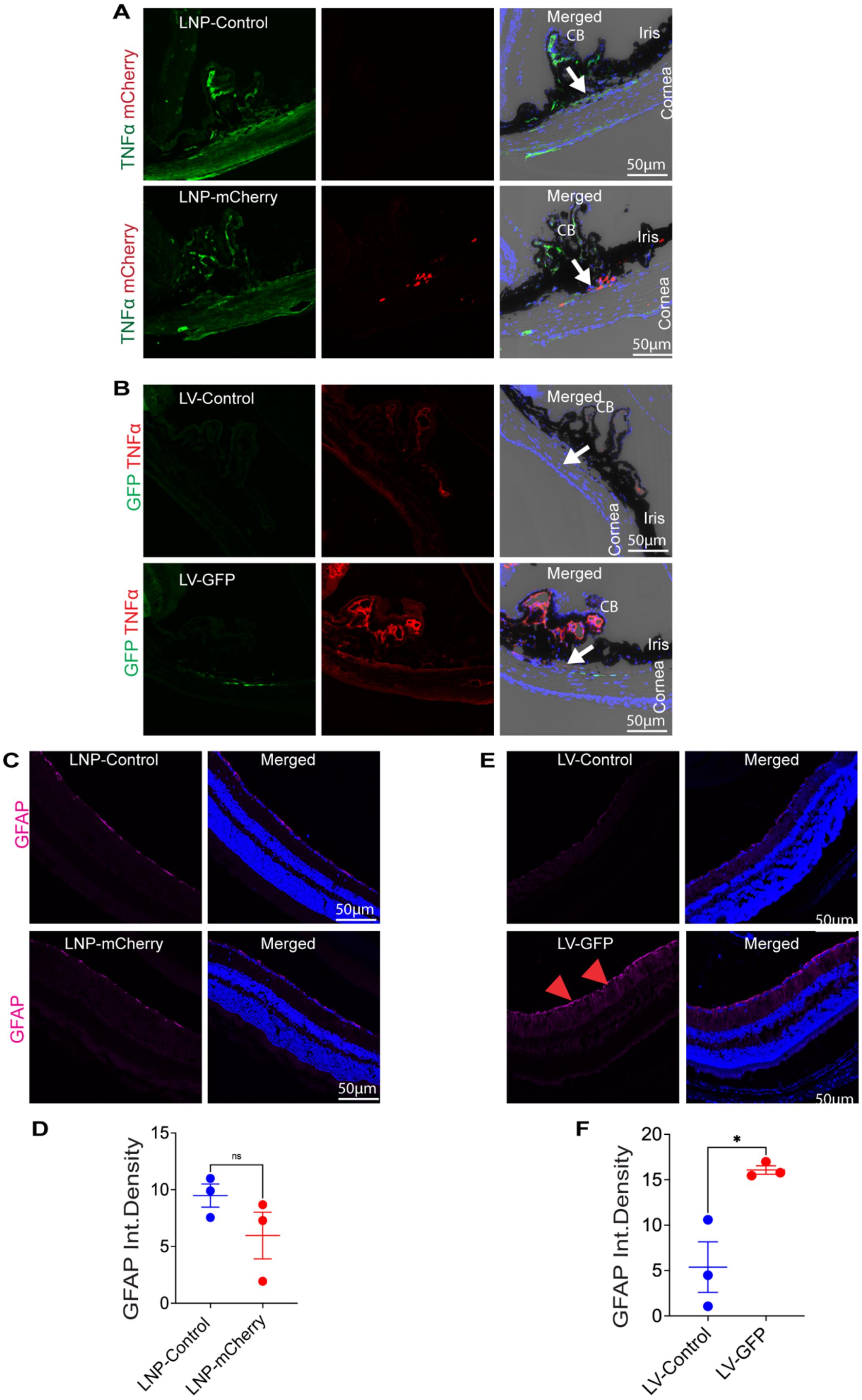
A
LNP-Control
Merged
CB
Iris
Cornea
50µm
TNFα mCherry
LNP-mCherry
B
LV-Control
LV-GFP
GFP TNFα
C
E
GFAP
LNP-Control
LNP-mCherry
LV-Control
LV-GFP
D
F
GFAP Int.Density
ns
*
15
10
5
0
20

Supplemental Figure S2. LNPs do not induce ocular inflammation or toxicity.
(A) Immunostaining of anterior segment sections for TNFα in eyes injected with LNP-mCherry showed no increase in inflammatory response compared with LNP-control; Scale bars: 50 μm, *n* = 3**.**
(B) In contrast, LV-GFP–injected eyes exhibited strong TNFα immunoreactivity in the anterior segment, including the TM region (arrows) and ciliary body; Scale bars: 50 μm, *n* = 3.
(**C–D**) Retinal cross-sections stained for GFAP revealed no elevation of GFAP expression in RGC layers following LNP-mCherry injection compared with control. Quantitative analysis confirmed no significant change in GFAP intensity; Scale bars: 50 μm, *n* = 3.
(**E–F**) In contrast, LV-GFP-treated retinas displayed a marked increase in GFAP expression in the RGC layer (arrowheads), consistent with a retinal inflammatory response. Statistical analysis confirmed a significant increase in GFAP intensity compared with LV-control; Scale bars: 50 μm, *n* = 3; (*p=0.0193, unpaired two-tailed t-test).

Together, these findings demonstrate that LNP delivery does not elicit ocular inflammation or retinal toxicity, in contrast to LV-based delivery, which triggered a robust inflammatory response.

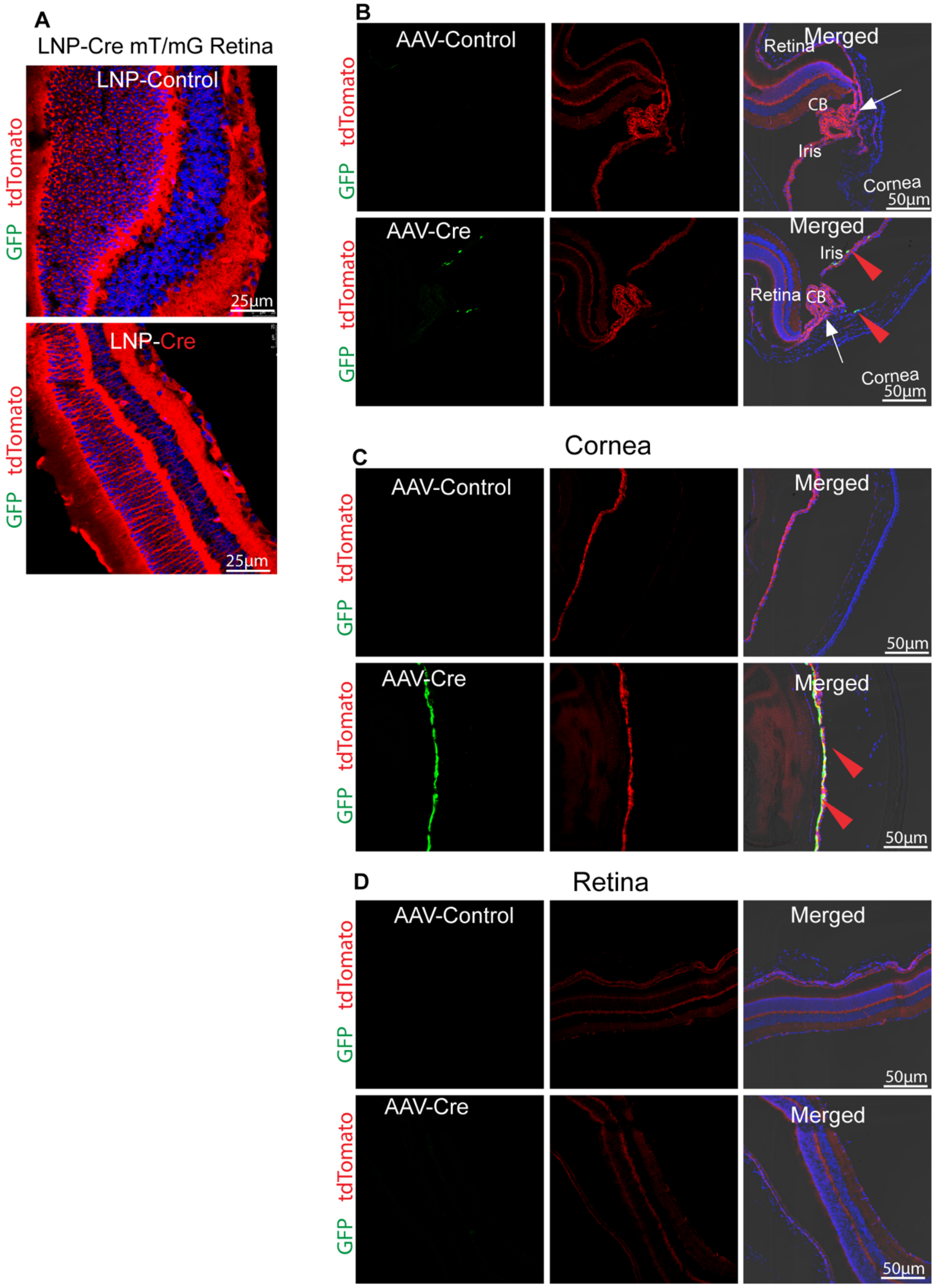
A
LNP-Cre mT/mG Retina
LNP-Control
LNP-Cre
GFP tdTomato
25µm
B
AAV-Control
AAV-Cre
Merged
Retina
CB
Iris
Cornea
50µm
C
Cornea
D
Retina

Supplemental Figure S3. Comparison of LNP-Cre and AAV-Cre tropism in mT/mG reporter mice.

(A) Retinal sections from *mT/mG* reporter mice injected with LNP-Cre showed no GFP signal, indicating that LNPs do not transduce retinal tissues compared withLNP-control; Scale bar: 50 μm, n=3.

(**B**) In contrast, cross-sections of *mT/mG* mice injected with AAV-Cre by single intracameral injection revealed Cre activity in multiple ocular tissues, including the iris and cornea (arrows), compared with AAV-control. Scale bar: 50 μm, *n* = 3.

(**C**) Higher-magnification cross-sectional analysis confirmed AAV-Cre transduction in the iris and corneal tissue. Scale bar: 50 μm, *n* = 3.

(**D**) Retinal sections demonstrated that AAV-Cre did not transduce the retina following a single intracameral injection. Scale bar: 50 μm, *n* = 3.

These findings highlight the ocular specificity of LNP delivery compared with the broader, non-specific tropism observed with AAV vectors.

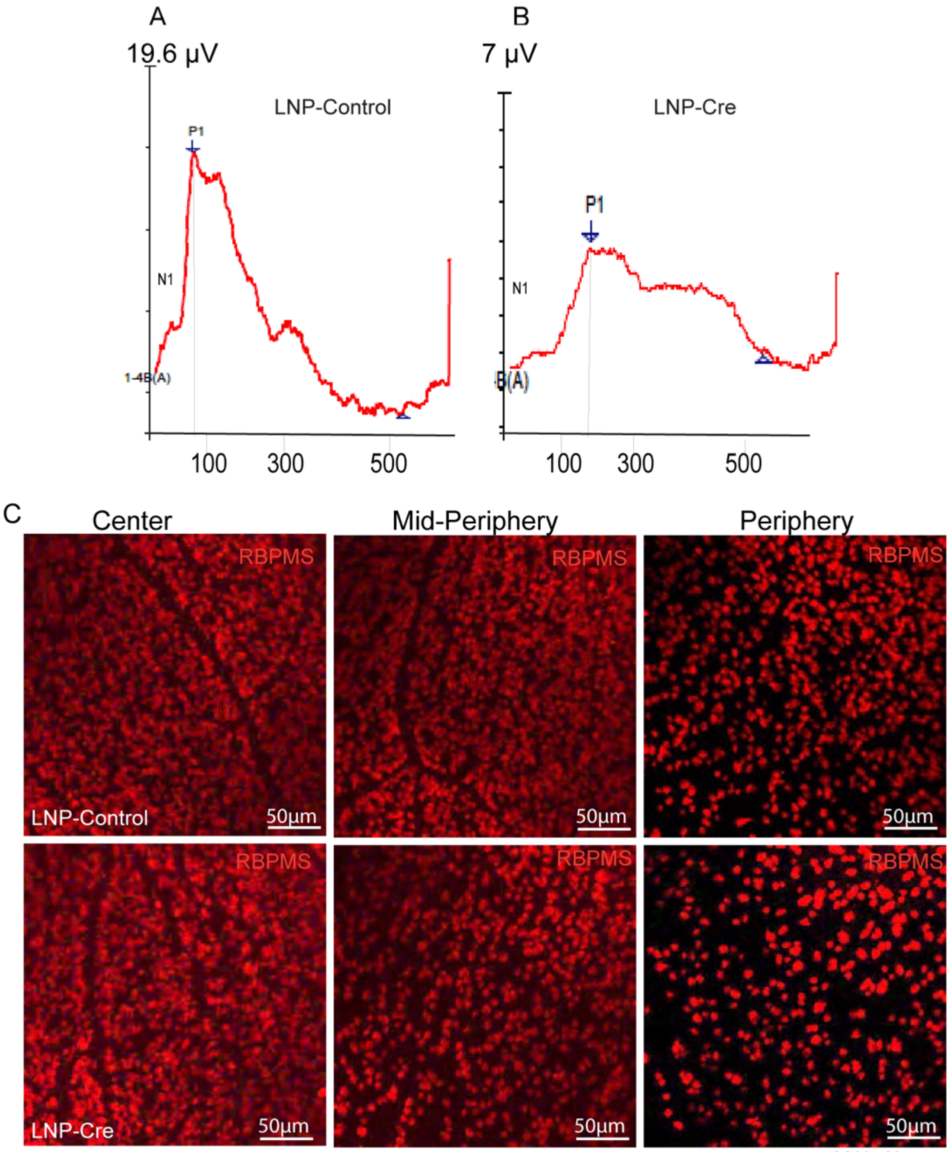

Supplemental Figure S4: IOP elevation in *Tg.CreMYOC^{Y437H}* mice leads to the loss of RGCs. Representative pattern electroretinography (PERG) waveforms from *Tg.CreMYOC^{Y437H}* mice treated with LNP-Control and LNP-Cre (A-B). Representative RBPMS staining of whole-mount retina, showing that LNP-Cre-mRNA-induced ocular hypertension in *Tg.CreMYOC^{Y437H}* mice lead to a significant decrease in the number of RGCs at the 15-week post-injection, compared to control retinas, particularly in the peripheral retina, a characteristic feature of POAG (D). Scale bar: 50 µm.

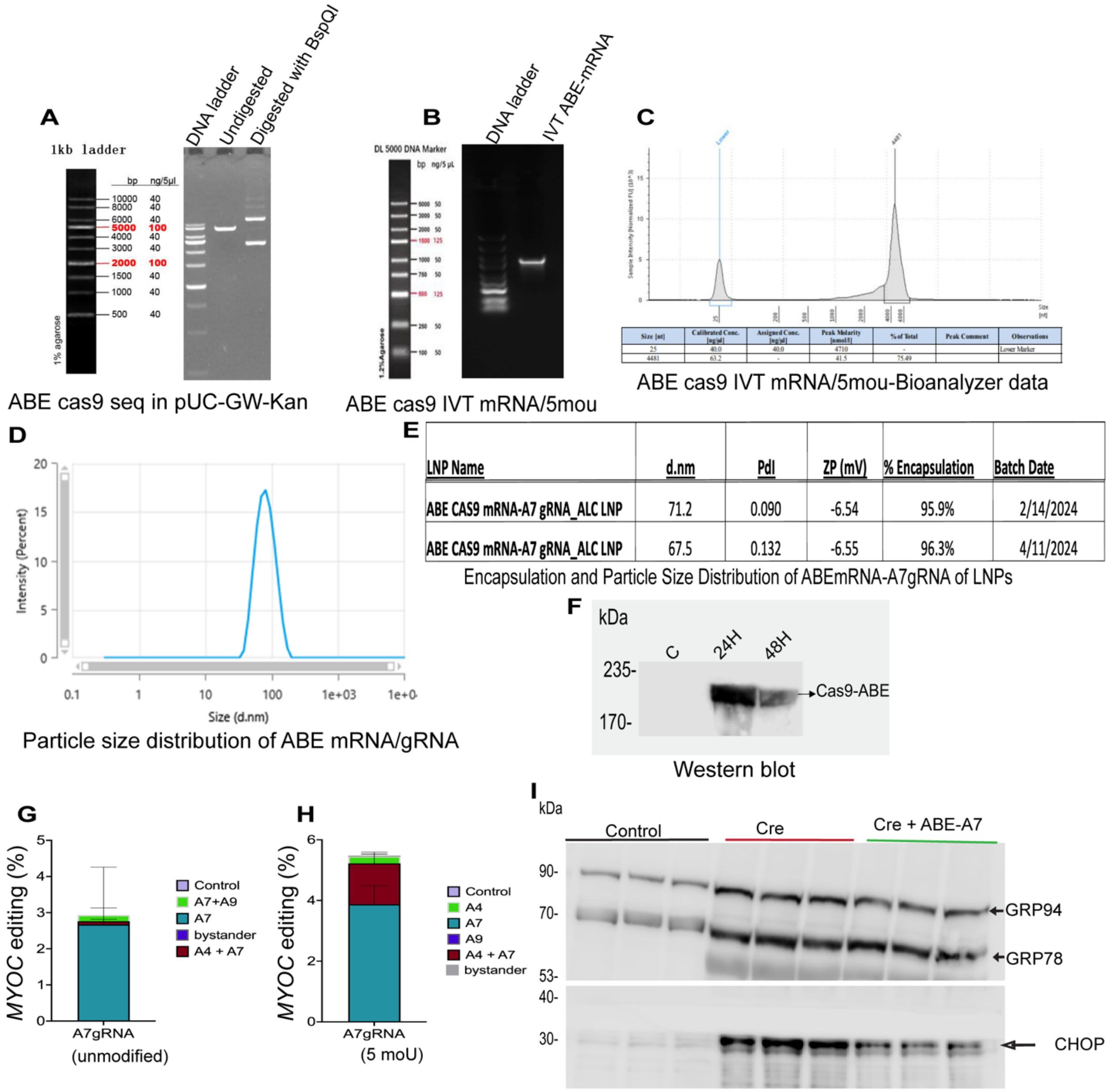

LNP Name	d.nm	PdI	ZP (mV)	% Encapsulation	Batch Date
ABE CAS9 mRNA-A7 gRNA_ALC LNP	71.2	0.090	-6.54	95.9%	2/14/2024
ABE CAS9 mRNA-A7 gRNA_ALC LNP	67.5	0.132	-6.55	96.3%	4/11/2024

Supplemental Figure S5: Characterization and expression of LNP-ABE-mRNA:

(**A**) The ABE gene was cloned into the pUC-GW-Kan plasmid, and its insertion was confirmed by restriction enzyme digestion, followed by analysis using agarose gel electrophoresis.

(B) A representative image of *in vitro*-transcribed ABE-mRNA analyzed by agarose gel electrophoresis shows a single distinct band aligned with the corresponding DNA ladder at the expected size.

(C) IVT ABE-mRNA purity analysis was performed using a bioanalyzer, which indicates >95% purity of the synthesized mRNA.

(D) Peak analysis of zeta potential and particle size distribution reveals that ABE-mRNA exhibits a uniform particle size with >95% distribution.

(E) LNP-ABE-mRNA was formulated with A7-gRNA, resulting in nanoparticles with an average particle size of 71.2 nm and >96% encapsulation efficiency.

(F) Western blot analysis of LNP-ABE-mRNA expression in TM3 cells. TM3 cells were transfected with LNP-ABE-mRNA and incubated for 24 hr and 48 hr. Western blot analysis demonstrates a robust Cas9-protein production within 24 hr, which had declined at 48 hr.

(**G-H**) NGS analysis of TM rings isolated from *Tg.CreMYOC*^{Y437H} mice injected with unmodified (**G**) or 5-moU-modified (**H**) LNP-ABE-mRNA showed 3.5% or 5.5% *MYOC* editing, respectively.

(**I**) Western blot analysis of ER stress markers demonstrated that LNP-ABE+A7 injected *Tg.CreMYOC*^{Y437H} mice exhibited reduced ER stress compared to both LNP-Cre–injected and control groups, as indicated by lower expression of KDEL and CHOP. These findings confirm that LNP-ABE+A7 treatment decreases mutant MYOC protein levels by alleviating ER stress.

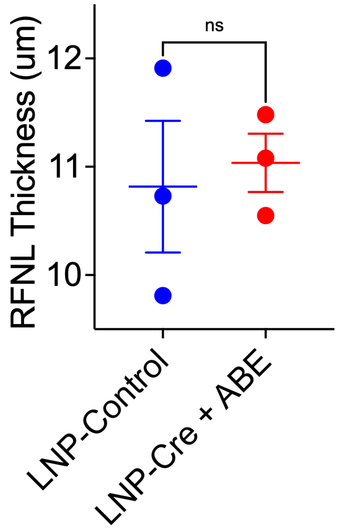

Supplemental Figure S6. OCT imaging of retinal segments showed no abnormalities in the retinal nerve fiber layer (RNFL) of eyes treated with LNP-ABE-A7 compared to control groups of *Tg.CreMYOC*^{Y437H} mice (n = 3 per group; ns = not significant, unpaired two-tailed t-test).

Table1:Guide RNA sequences targeting MYOC.

MYOC.A9.gRNA	AAGCTAGCATGAGGTTCTTCGTTTTAGAGCTAGAAATAGCAAGTTAAAATAAGGCTAGTCCGTTATCAACTTGAAAAAGTGGCACCGAGTCGGTGC
MYOC.A7.gRNA	GCTAGCATGAGGTTCTTCTGGTTTTAGAGCTAGAAATAGCAAGTTAAAATAAGGCTAGTCCGTTATCAACTTGAAAAAGTGGCACCGAGTCGGTGC
