# Supplementary material for: Lipid-nanoparticle-mediated base editing of the trabecular meshwork rescues glaucoma in vivo
Source: JCI Insight. 2026 Feb 9;11(3):e195593. doi: 10.1172/jci.insight.195593 (PMC12892903; doi:10.1172/jci.insight.195593)

ABE-mRNA expression in TM cells

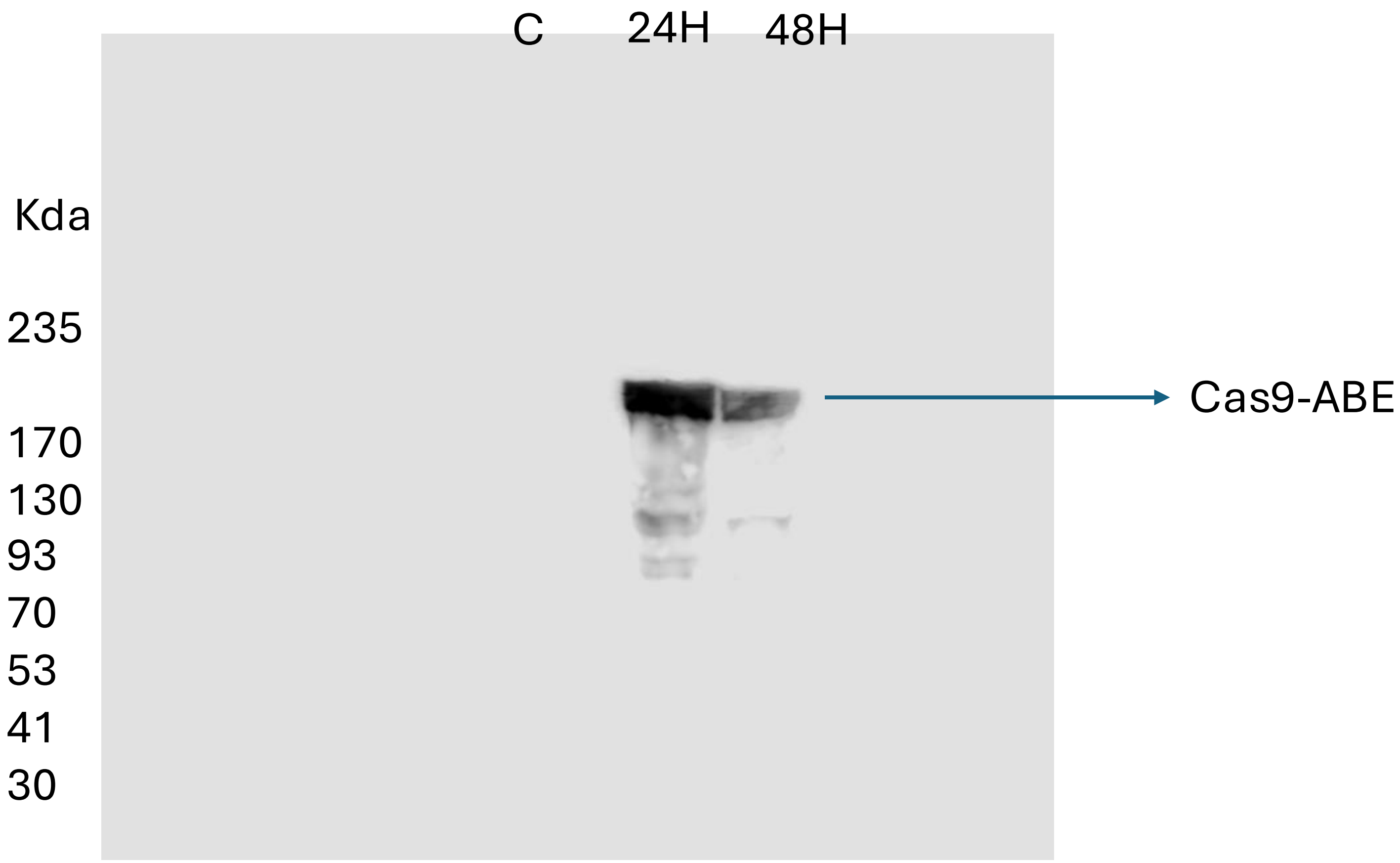

Western blot analysis

MYOC editing in ABE-A7 treated **TG.MYOC^{Y437}** mice

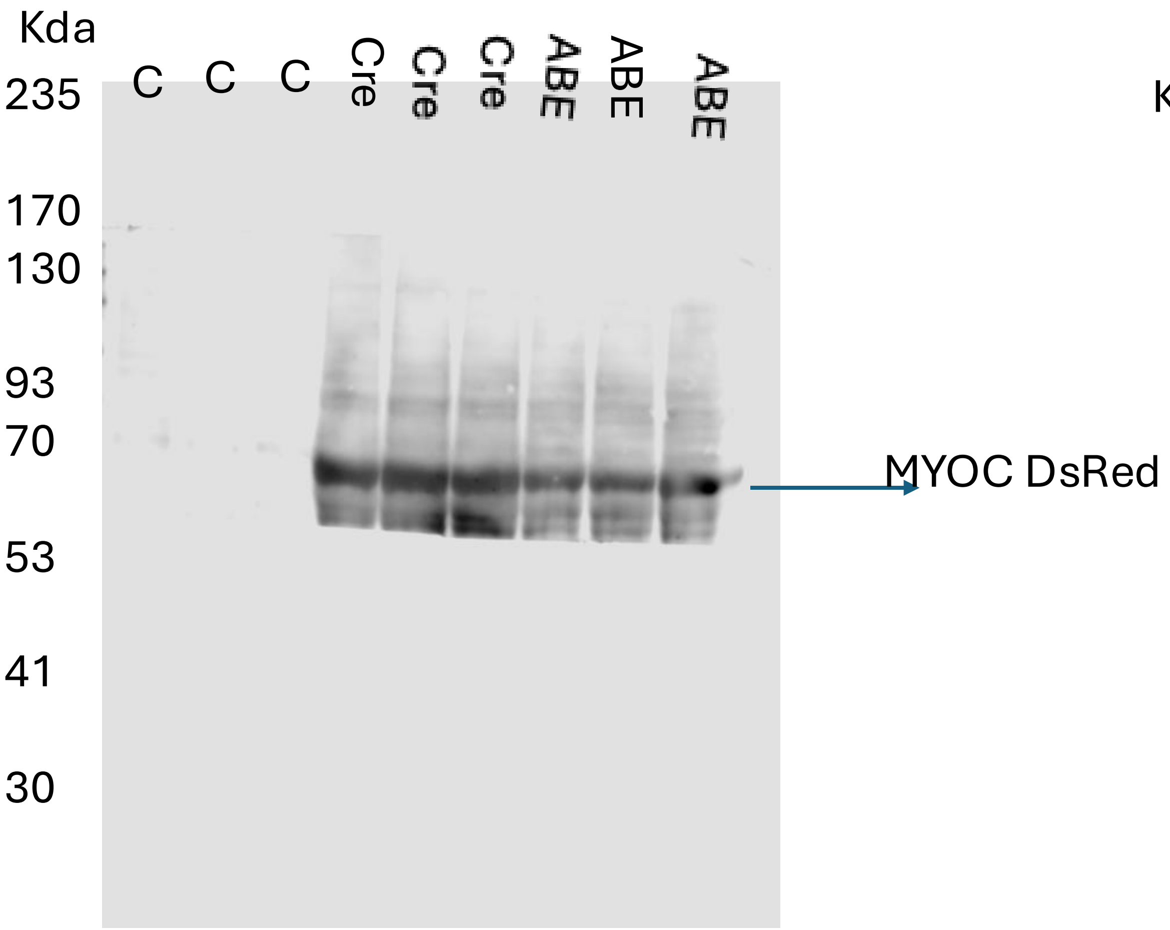

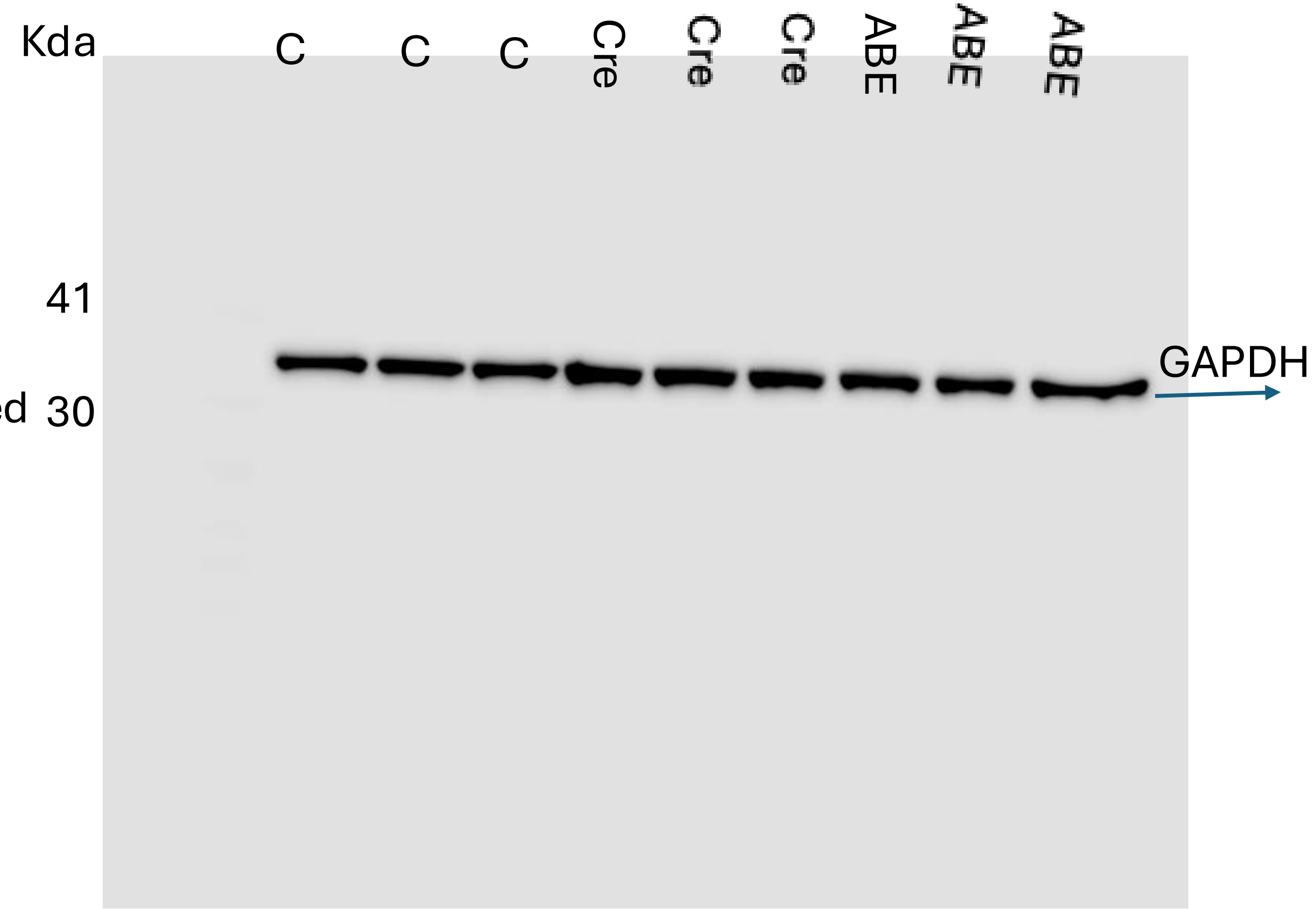

ER Stress analysis by KDEL and CHOPin ABE-A7 treated TG.MYOC^{Y437} H mice

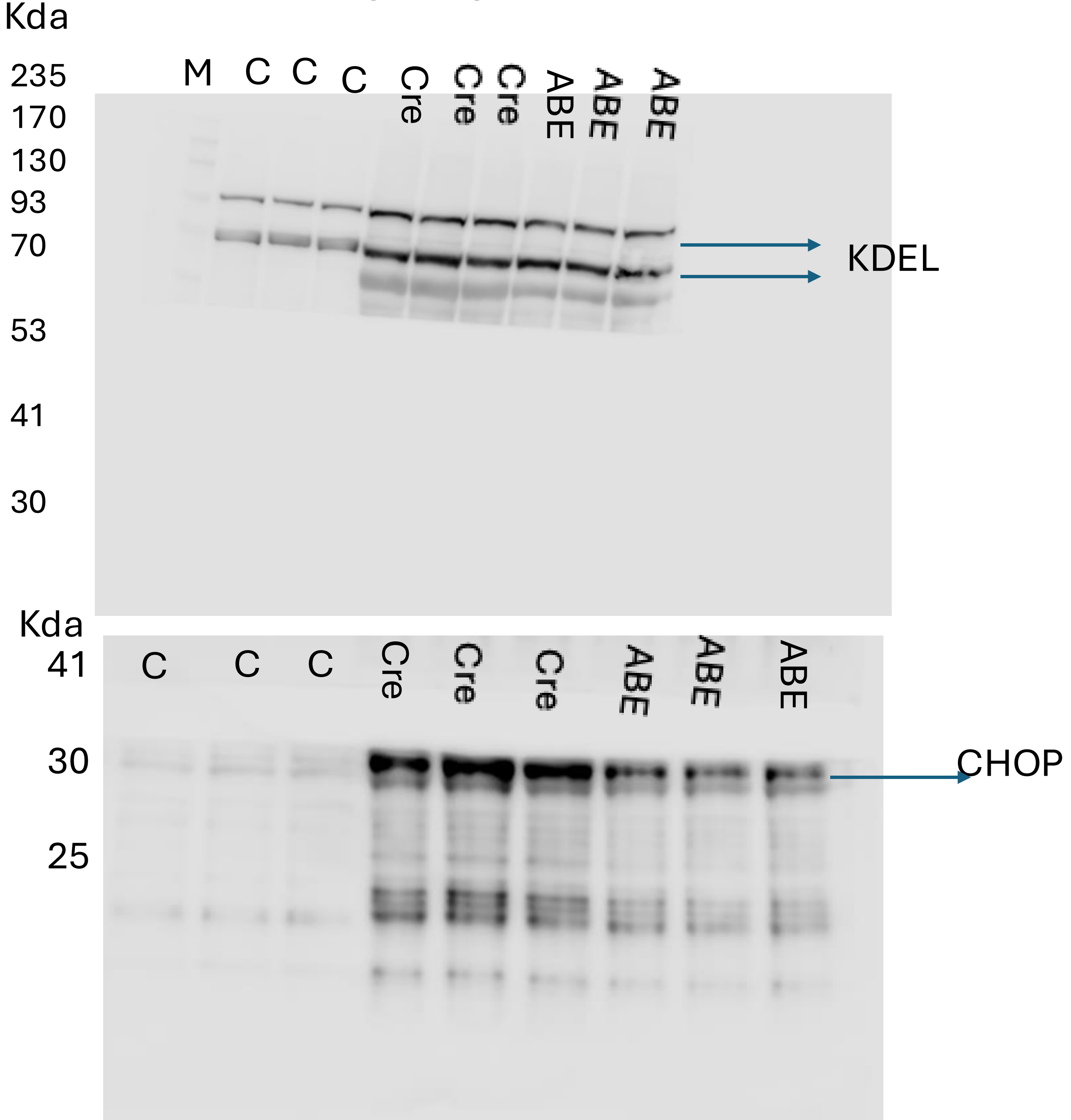

Supplement: Unedited blot and gel images [file jciinsight-11-195593-s093.pdf]
